# Chromosomal-Level Whole Genome Assemblies of *Crassostrea Virginica*

**DOI:** 10.64898/2026.10.13.758928

**Published:** 2026-10-19

**Authors:** Liam Cawood, Shiying Wang, Eli Kleiman, Zhiyuan Chen, Isabel Kleiman, Nicole Fair, Ruby Brownell, Irina Tikhonova, Zicheng Li, Josephine Hoh

**Affiliations:** 1 Department of Environmental Health Sciences, School of Public Health, Yale University, New Haven, CT 06510, USA; 2 Department of Genetics and Pediatrics, School of Medicine, Yale University, New Haven, CT 06510, USA; 3 Department of Chronic Disease Epidemiology, School of Public Health, Yale University, New Haven, CT 06510, USA; 4 Department of Ophthalmology, School of Medicine, Yale University, New Haven, CT 06510, USA; 5 Center of Genome Analysis, Yale University, West Haven, CT 06516, USA; 6 Department of Applied Mathematics, Yale University, New Haven, CT 06520, USA

## Abstract

The eastern oyster, *Crassostrea virginica*, is an ecologically important foundation species and a valuable aquaculture organism. To support genomic studies and future breeding applications, we generated PacBio HiFi, Illumina, and Hi-C sequencing data from wild adult oysters, collected from Long Island Sound and assembled a chromosome-level reference genome. The final assembly spans 529.0 Mb and consists of 10 chromosomes with a scaffold N50 of 55.46 Mb and a contig N50 of 16.0 Mb. The resulting reference genome achieved 99.7% genome completeness and reduced duplicated universal single-copy ortholog content from 18.7% to 1.1%. Genome annotation identified 33,259 protein-coding genes and revealed that 43.2% of the genome is composed of repetitive elements. All sequencing data underwent stringent quality control, and the assembly results were validated through multiple technical evaluations. These dataset and resources of oyster genomics provided future studies such as effective breeding and sustainable farming.

## Background & Summary

Marine invertebrates thrive in highly dynamic coastal environments, yet the genomic basis of their resilience remains poorly understood. Understanding how these organisms maintain homeostasis under environmental extremes provides broader insights into cellular stress tolerance, innate immunity, and genome plasticity across animals. The eastern oyster (*Crassostrea virginica*) is native to the Atlantic coast of North America and plays both major economic and ecological roles. Oyster reefs stabilize shorelines, provide habitat for diverse species, and improve water quality through filter-feeding^1–3^. Because of these ecosystem services, *C. virginica* has become both a keystone species in coastal restoration and a cornerstone of aquaculture along the Atlantic seaboard. Its dual ecological and economic importance makes it a rare case where understanding genome resources directly informs conservation biology and sustainable food production. Moreover, as a sentinel for coastal health, the oyster’s physiological responses to salinity, temperature, hypoxia, and pathogens offer readouts of environmental change that are relevant to ecosystem management and public health^4–7^.

As a sessile filter feeder, *C. virginica* endures large fluctuations in salinity^8,9^, temperature^10,11^, oxygen^12,13^, and pathogen load^6,7,14^. Understanding the genomic architecture underlying environmental response provides insights into how eukaryotic genomes coordinate regulatory plasticity and gene family diversity to sustain organismal resilience. Decades of selective breeding and accumulating genomic resources have further established *C. virginica* as a model for stress adaptation and environmental genomics^15–17^, yet fully resolving genome structure and gene family organization requires an accurate and complete reference genome.

The existing assembly (*C. virginica*-3.0) contains haplotig redundancy and chromosomal misassemblies ^18^ and subsequent distorting cross-species comparisons^19^. With growing interest in comparative genomics across oysters, such as *Magallana gigas*, *M. hongkongensis*, and *Ostrea edulis^20–22^*, a structurally accurate *C. virginica* genome is essential for reliable evolutionary comparisons. This species occupies a pivotal position within the Crassostreinae subfamily, having diverged from Pacific species over 80 million years ago^23^. This deep evolutionary split, combined with the species’ resilience to fluctuating estuarine environments, makes *C. virginica* an important system for studying genome evolution in marine bivalves.

To improve genomic resources for this species, we generated PacBio-HiFi, Illumina whole-genome sequencing (WGS), and Hi-C data and produced a chromosome-level genome assembly of *C. virginica* (Fig. 1). The final assembly spans 529.0 Mb across 10 chromosomes and consists of 81 contigs and 71 gaps, with a scaffold N50 of 55.3 Mb and a contig N50 of 16.0 Mb. Assembly statistics at each stage of the workflow are summarized in Step-by-step metrics tracking across the assembly workflow (Table S4 and SI-04).

Genome annotation was performed to characterize sequence composition and gene content (Fig. 2). Illumina WGS coverage across the genome was uniform (mean=30.8x, SD=±7.74x), with only 0.04% of windows deviating more than two standard deviations from the genome-wide mean. Repeat annotation identified 43.2% of the genome as repetitive, including DNA transposons (7.1%), LTR retrotransposons (7.1%), LINEs (3.4%), and SINEs (3.6%) (Table S1). Telomeric repeat arrays were detected near the ends of six chromosomes (Table S2), and putative centromeric repeat arrays were identified on seven chromosomes (Table S3). Gene prediction identified 33,259 protein-coding genes (Fig. 2B; Data S1), of which approximately 68% received functional annotation (Fig. 2C; Data S1). Genome-wide distributions of gene density, transposable elements, and segmental duplications are summarized in Fig. 2D–F.

We further examined the removed sequences to determine whether any annotated genes were lost or were redundant with retained regions (Methods). The analyses showed that all 440 gene predictions identified on the removed sequences were represented in the retained Stage 4 assembly. Of these, 391 had corresponding matches in the Stage 4 predicted proteome, while the genomic DNA underlying the remaining 49 predictions was retained in Stage 4 at high sequence identity and coverage. The latter differences are therefore attributable to differences in gene prediction rather than physical loss of the corresponding genomic regions. Thus, we found no evidence that manual haplotig removal resulted in the loss of unique protein-coding gene content (Table S4).

## Methods

### Sample Collection and Data Generation

Adult *Crassostrea virginica* individuals were collected from a commercial oyster farm in Long Island Sound, New York. Immediately after harvesting, specimens were placed on ice, transported to the laboratory, shucked, and whole tissues were flash-frozen in liquid nitrogen. Genomic DNA was extracted using E.Z.N.A. Mollusc & Insect DNA Kit, and quality was assessed with NanoDrop spectrophotometry.

Hi-C libraries were prepared with the Arima-HiC Kit according to the manufacturer’s protocol and sequenced on an Illumina NovaSeq X platform. Illumina whole-genome sequencing (WGS) libraries were prepared in parallel and sequenced on the same platform. High-molecular-weight DNA was also used to generate PacBio HiFi reads on the Revio system, following the standard PacBio protocol. Read depths, counts and N50 information were provided in SI-02 (Table S7).

### Genome Assembly and Quality Control Assessment

A workflow of de Novo Assembly was provided (Fig 1A, SI-01)

We conducted genome assembly using raw data from PacBio HiFi long reads, Illumina whole-genome reads, and Arima Genomics Hi-C data. Raw Illumina reads were first assessed with FastQC v0.11.9 and adapter- and quality-trimmed using Trimmomatic v0.36 (Q30 cutoff)^24^.

PacBio HiFi reads were used directly without trimming. Primary contigs were assembled with Hifiasm v0.24.0 ^25^ using default parameters. To reduce allelic redundancy, one round of Purge_Dups v1.2.6 (default) ^26^was applied to the contig assembly, guided by Illumina and HiFi read depth profiles.

Chromosome-scale scaffolding was performed on Hi-C data. Trimmed Hi-C reads were aligned to contigs with BWA-MEM v0.7.17^27^, and scaffolding was conducted with Juicer v2.0.1 ^28^ and the 3D-DNA pipeline (2021-Mar-18 version) ^29^under default parameters. Manual curation and misjoin correction were performed with Juicebox Assembly Tools v2.15. After scaffolding, residual haplotigs and unresolved heterozygous regions were identified using a two-step selfalignment approach. Whole-genome self-alignment was first performed with minimap2 v2.22 ^30^ and then confirmed with MUMmer4 v4.0.1^31^ (“nucmer --maxmatch -c 100 -b 500 -l 50”).

Candidate redundant regions were identified based on both alignment and coverage criteria. Coverage analysis - a mean depth of 30.8× (SD = 7.74×) was calculated from duplicate-removed, MAPQ ≥30 filtered alignments in 100-kb windows. Non-redundant alignments were filtered to retain hits with alignment length ≥10kb and identity ≥95%. Illumina WGS coverage was calculated in 100kb bins, and half-coverage zones were defined as bins with coverage <65% of the genome-wide mean. Nearby half-coverage zones within 400kb were merged to delineate candidate heterozygous regions. A scaffold was considered for manual removal only if the residual haplotig segment was ≥500kb in length. Using this workflow, approximately 3.0Mb of residual haplotigs or unresolved heterozygous regions were manually removed, and scaffold breakends were stitched with 100 Ns at each join to represent unknown gap sequences, yielding the final non-redundant assembly. All remaining high-confidence self-alignments were classified into three categories based on their genomic positions: tandem duplications, where duplicated segments were separated by <100kb; segmental duplications, where duplicated segments were separated by >100kb on the same chromosome; and inter-chromosomal segmental duplications, where duplicated segments resided on different chromosomes.

Assembly quality was assessed using multiple complementary approaches. BUSCO v5.8.2^32^ with the mollusca_odb10 dataset evaluated gene completeness. Quast v5.3.0^33^ reported standard assembly statistics, including N50, L50, total size, and GC content. Merqury v1.4.1^34^ was used for 21-mer-based quality value estimation, with k-mer databases generated using Meryl v1.4.1 from both PacBio HiFi and Illumina WGS reads. Whole-genome structural consistency was further evaluated by comparing the new assembly to the previous *C. virginica_3.0* reference. Pairwise genome alignments were generated using MUMmer4 v4.0.1 (“nucmer --mum -c 100 -b 500 -l 50”), and alignment results were manually filtered to retain alignments ≥ 0.01Mb (10kb) and ≥ 90% identity. These filtered alignments were visualized as whole-genome dot plots, which revealed collinear chromosomes, scaffold-level misorientations, and large mis-joints present in the previous assembly.

Haplotig purging and redundancy removal constituted a central component of this study. To ensure reproducibility and transparency he assembly workflow (SI-01) and associated quality-control procedures were provided with a detailed description as follows:

The built-in purge function in Hifiasm was enabled using the default settings. One round of Purge_Dups was performed on the primary contig assembly to remove residual haplotypic duplications ^26^ https://github.com/dfguan/purge_dups/issues/50.

Purge_Dups was applied before Hi-C scaffolding. We followed the established standard: Purging: Uncollapsed and duplicate haplotypes are purged from the draft assembly. Scaffolding: The cleaned sequences are then passed to Hi-C scaffolding tool, 3D-DNA, to orient and order them into chromosomes.

Applying Purge_Dups to the initial contigs allows us to identify and remove haplotypic duplications and heterozygous overlaps. This cleanup step prevents downstream scaffolding tools from making false joins, generating a much cleaner sequence prior to chromatin conformation mapping ^35,36^.

Manual haplotig removal involved the following steps in our assembly: a) inspecting read-depth histograms for a bimodal distribution and removed contigs with low coverage, b) Contigs were examined for similarities via pairwise alignment (the minimap2 tool). We removed redundant haplotigs for those short contig aligned to a long and primary contig with more than 95 per cent similarities, c) we used BUSCO for reasonable, instead of high numbers, of single-copy ratio to ensure the genomic region is not duplicated, d) We also used long-range scaffolding data, Hi-C, removing those haplotigs which present unexplained several paths on chromosomes.

### Repetitive Element and Tandem Array Annotation

Repetitive elements in the C. virginica genome were identified entirely through de novo discovery. RepeatModeler v2.0.7 ^37^ was used to generate a species-specific repeat library. The assembly was then soft masked with RepeatMasker v4.1.8 ^38^ using only the custom de novo library, classifying transposable element (TE) into major categories; including DNA transposons, LINEs, SINEs, and LTR retrotransposons. For quantitative analyses, overlapping TE annotations of the same class were merged into non-overlapping intervals to avoid double-counting bases covered by nested or partially overlapping repeats. Genome-wide TE content was calculated per chromosome and in 200kb non-overlapping windows, generating base-level coverage estimates for visualization and for correlation analyses with gene and duplication density.

Telomeric repeats were identified using Tandem Repeats Finder (TRF) v4.09.1^39^ with parameters “2 7 7 80 10 50 2000”. Candidate telomere tracts were defined as “TAAGGG” hexamer arrays of ≥60 bp, located within 10kb of chromosome ends, and showing ≥95% sequence identity.

Candidate centromeric arrays were extracted from the TRF output using the following filters: total array length ≥10kb, repeat unit (period) ≥150 bp, copy number ≥10, and percent match ≥ 75%. The basic repeat units of the filtered arrays were then compared using BLASTN v2.13.0+^40^ (-evalue 1x10^−5^), and hits were retained if identity was ≥80% and aligned length ≥70% of the smaller array. Arrays passing these criteria were clustered into families by connecting arrays as nodes in an undirected graph when a qualifying BLASTN hit existed between them. These graph-based clusters were then manually reviewed to confirm family assignments and calculate centromere indices for each chromosome, which were compared against expected positions from prior *C. virginica* cytogenetic studies. Arrays forming family-specific clusters in chromosomal interior regions were annotated as putative centromeric repeats.

### Gene Prediction and Functional Annotation

Protein-coding genes were predicted using RNA-seq–guided annotation. Publicly available *C. virginica* RNA-seq datasets from 10 samples spanning mantle, gonad, and mixed tissues from both sexes were preprocessed with Fastp v0.20.0 ^41^ (“--cut_front --cut_tail --detect_adapter_for_pe --cut_mean_quality 20 -l 50”). Filtered reads were aligned to the genome using STAR v2.7.11b ^42^ in two-pass mode to improve splice junction detection. All alignments were then merged with Samtools v1.6 ^43^ to generate a unified BAM file for gene model training.

Gene structure prediction was performed using BRAKER v3.0.8^44^, which integrates RNA-seq alignments with ab initio modeling to generate high-confidence gene models. Coding sequences (CDS) and predicted proteins were extracted using GffRead v0.12.7 ^45^ to produce FASTA files for downstream analysis. Functional annotation was conducted with EggNOG-mapper v2.1.12^46^, which assigned orthology-based functional descriptions, putative gene names, GO terms^47^, and KEGG pathways^48^.

To assess potential errors in gene loss/gain, we performed comparative genomic analysis by aligning the assembled genomes against five oyster species’ reference genomes. Gene loss/gain might be computationally validated if the conserved regions were missing or enriched. In the context of evolution, we conducted comparative chromosome-level analyses against five species, including O. edulis (xbOstEdul1.1), M. gigas (xbMagGiga1.1), M. angulata (ASM2561291v2), M. ariakensis (ASM4011448v1) and M. hongkongensis (ASM1577677v1). We found that all ten chromosomes corresponded to homologous chromosomes in a one-to-one fashion across the five species. While overall global structures seem to be conserved across species, divergence is revealed at finer genomic scales. Rather than large-scale chromosomal reshuffling, most structural differences are driven by intrachromosomal remodeling, such as local rearrangements, insertions, and inversions that are unevenly distributed. Gene loss/gain error might be computationally validated if the conserved regions were missing or enriched. However, our comparative analyses against the Magallana clade (Pacific oyster species) showed reduced ortholog conserved regions but enriched in *C. virginica*, containing immune and stress-related genes.

### Technical Validation

The quality and structural accuracy of the *Crassostrea virginica* genome assembly were evaluated using multiple complementary approaches. Genome completeness was assessed using BUSCO v5.8.2 with the mollusca_odb10 dataset. The assembly achieved 99.7% completeness and showed a substantial reduction in duplicated single-copy orthologs compared with the existing reference assembly (*C. virginica*-3.0), where BUSCO duplication decreased from 18.7% to 1.1%.

Consensus sequence accuracy was evaluated using k-mer–based analysis with Merqury v1.4.1. A 21-mer database was constructed using both PacBio HiFi reads and Illumina whole-genome sequencing reads. Comparison between sequencing-derived k-mers and the assembled genome supported high consensus accuracy of the assembly.

K-mer analysis confirmed high consensus accuracy of the assembly. The Merqury QV score analyses were done chromosome-by-chromosome for 10 chromosomes, with separate WGS and PacBio datasets. PacBio HiFi reads averaged QV score 69.63, and Illumina reads averaged 43.97 (Fig. 1B). Compared with the previous reference (*C_virginica*-3.0), the new assembly shows improved contiguity and reduced genome redundancy, with BUSCO duplication reduced from 18.7% to 1.1% while achieving 99.7% gene completeness (Fig. 1C). Hi-C contact maps support chromosome-scale scaffolding, showing strong intra-chromosome interaction signals and no evidence of major inter-chromosomal misjoins (Fig. 1D). Whole-genome alignment and synteny comparisons further illustrate structural consistency between assemblies and highlight corrections to structural inconsistencies present in the previous reference (Fig. 1E–F). A summary of sequencing specifics can be found in supplemental information (Table S7).

Uniformity of genome representation was evaluated using Illumina WGS read coverage across the genome. Coverage analysis showed a mean depth of 30.8x with a standard deviation of ±7.74x. Only 0.04% of genomic windows deviated by more than two standard deviations from the genome-wide mean, indicating consistent coverage and the absence of large collapsed or overrepresented regions.

Structural consistency was further evaluated by aligning the assembly to the previous *C. virginica*-3.0 reference genome using whole-genome alignment with MUMmer4. The resulting dot plots show largely collinear chromosomes and highlight structural inconsistencies present in the earlier assembly.

In addition, we performed a comprehensive contamination assessment (SI-03) using three complementary approaches.

First, the assembled genome was evaluated using the NCBI Foreign Contamination Screen (FCS-GX), which identified no sequences requiring removal.

Second, GC content versus sequencing coverage analyses were performed independently using both Illumina WGS and PacBio HiFi reads, showing a single, tightly clustered distribution with no evidence of secondary clusters indicative of contaminating sequences. The only outlier corresponded to a multi-copy riboSIal DNA (rDNA) region, as confirmed by BLAST analysis.

Third, we evaluated taxonomic classification using Kraken2. However, because the current Kraken2 database does not include the *C. virginica* reference genome, oyster-derived sequences cannot be reliably assigned to the correct species, making Kraken2 unsuitable for assessing contamination in this assembly.

Together, the FCS-GX and GC-coverage analyses provide no evidence of substantial contamination in the final genome assembly.

These validation analyses support the quality and structural consistency of the chromosome-level *C. virginica* genome assembly.

### Usage Notes and Limitations

This chromosome-level assembly presented an improved version of the Crassostrea virginica whole genome, compared with the previous reference genomes. The assembly, sequencing datasets, and annotation files can provide resources for breeding strategies using genomic approaches.

Several imitations in the reference genome assembly will still need to be considered or improved in the future for completeness as well as diversity. This assembly was on based on one oyster; thus, it lacked the information of population diversity including insertions, deletions, or structural variants (copy number variations) in individuals. Repetitive DNA regions were difficult to resolve by current sequencing technologies. Since oysters’ genomes are diploid, the haplotigs often represented their distinct maternal and paternal chromosomes. At this assembly we purge all haplotigs and information could be lost for true heterozygosity.

## Supplementary Material

Supplement 1

Supplement 2

Data S1, making the BRAKER annotation available, consists of the following four files

braker.gff3: full BRAKER gene annotation in GFF3 format (containing 33,259 predicted genes)

cds.fa: predicted coding-sequence FASTA

proteins.fa: predicted-protein FASTA

README.txt: file descriptions and assembly information

The annotation package has been deposited in Zenodo and assigned the persistent DOI: https://doi.org/10.5281/zenodo.22014057 DOI: 10.5281/zenodo.22014057

## Figures and Tables

**Figure 1.**
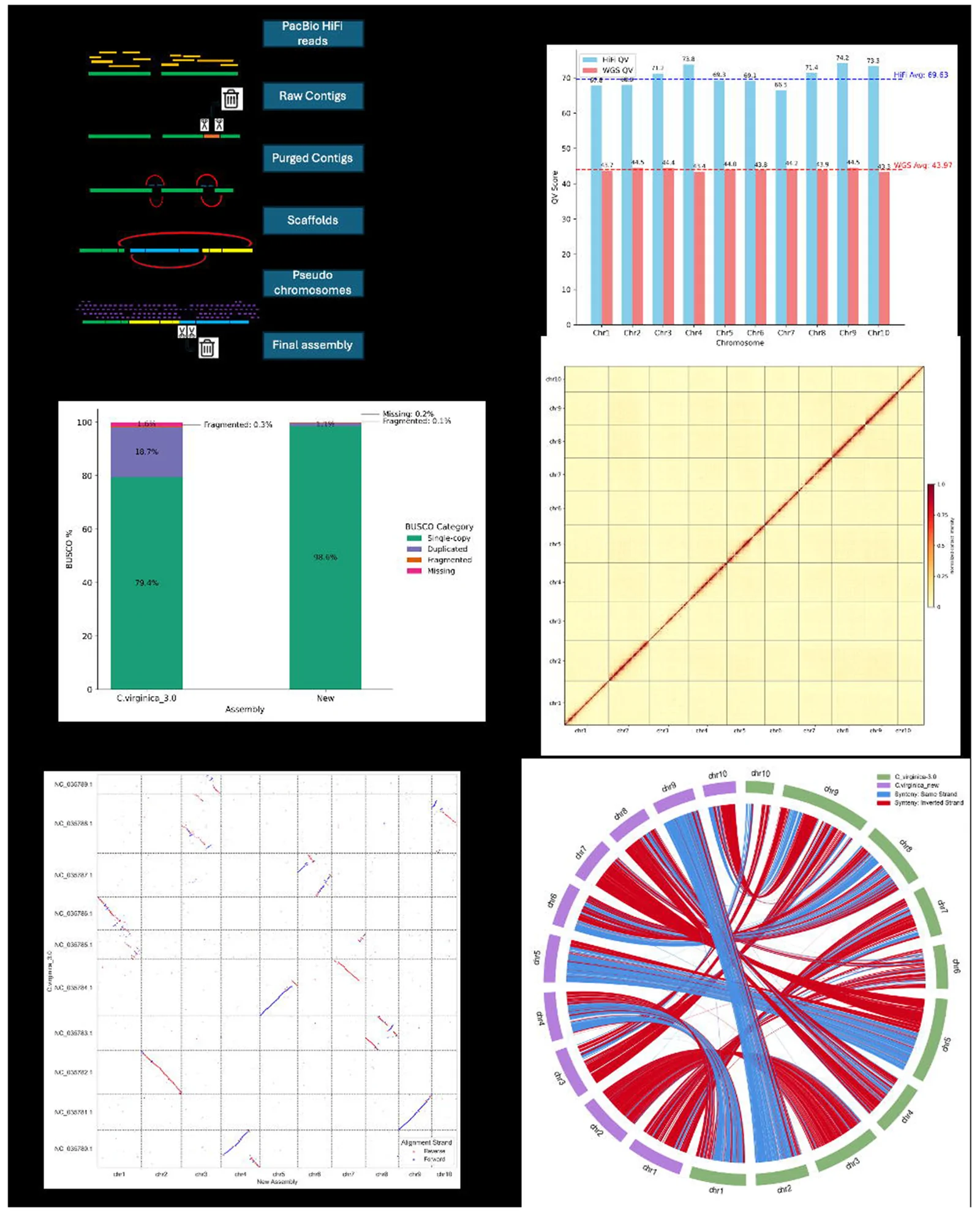
Overview data generation and validation of the chromosome-level assembly of Crassostrea virginica. (A) Schematic overview of the genome assembly workflow. (B) Per-chromosome 21-mer quality value (QV) scores estimated from PacBio HiFi and Illumina whole-genome sequencing (WGS) reads. (C) BUSCO gene completeness assessment using the mollusca_odb10 dataset. (D) Hi-C contact map illustrating chromosome-scale interaction patterns and contig scaffolding. (E) Whole-genome dot plot alignment (≥10 kb alignment length, ≥90% sequence identity) between the new assembly and the previous C. virginica-3.0 reference genome. (F) Genome-wide synteny chord diagram showing chromosomal correspondence between the two assemblies.

**Figure 2.**
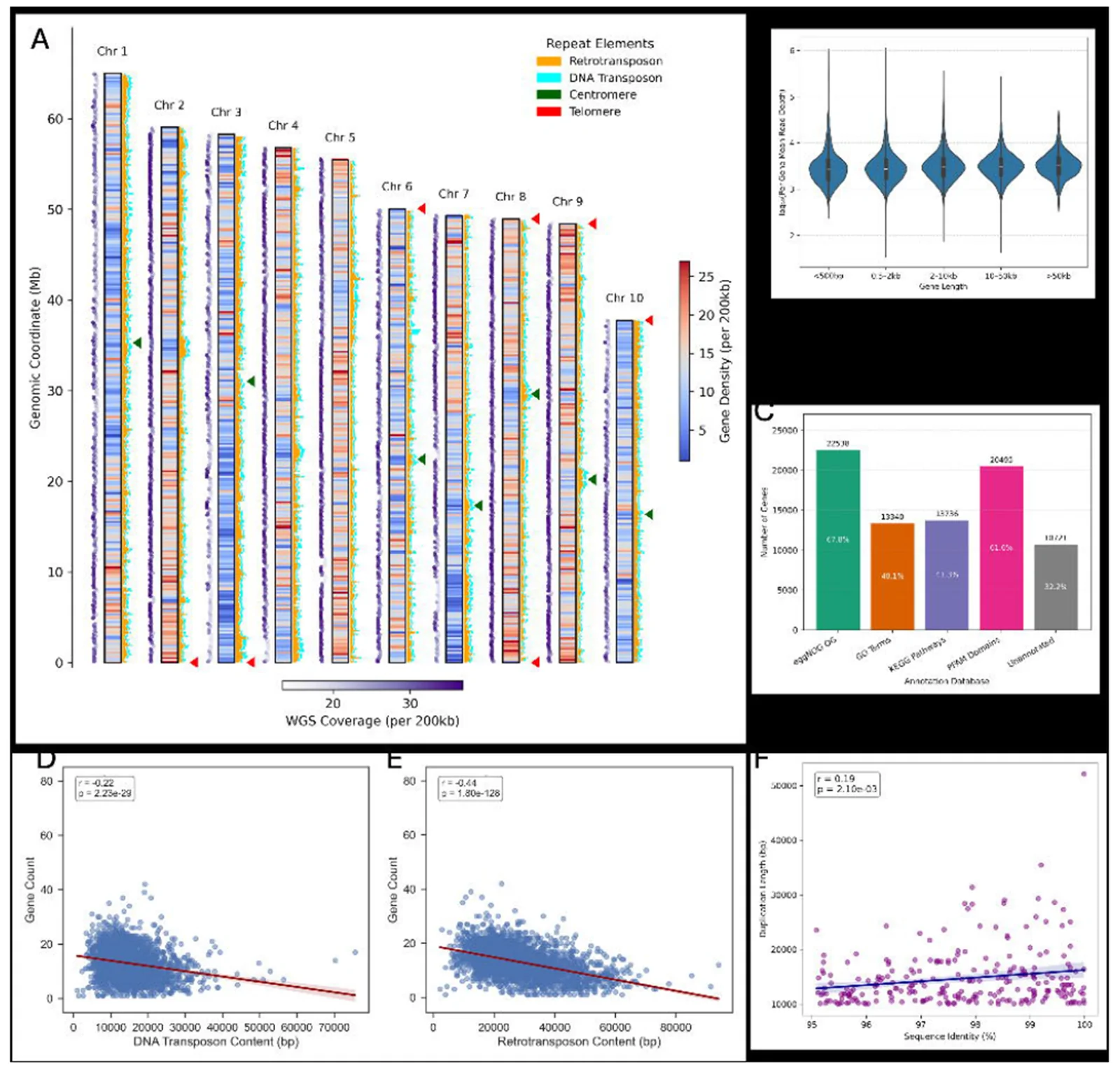
Genome annotation and structural features of the C. virginica genome. (A) Chromosomal landscape of gene density, repeat content, and sequencing coverage across the 10 chromosomes. Each vertical bar represents a chromosome scaled by length. The internal heatmap shows gene density per 200 kb window. Whole-genome sequencing (WGS) coverage is displayed as a gradient to the left of each chromosome, and transposable element content per 200 kb window is shown as stacked bars on the right. Putative telomeric and centromeric repeat arrays are indicated by triangles. (B) Violin plot showing RNA-seq read depth supporting predicted gene models across gene length bins. (C) Functional annotation summary of predicted protein-coding genes, including eggNOG orthogroup assignments, Gene Ontology (GO) terms, KEGG pathways, and PFAM domains. (D–E) Scatterplots showing the relationship between gene density and transposable element (TE) content across 200 kb genomic windows. (F) Scatterplot showing the relationship between duplication length and sequence identity among large segmental duplications (≥10 kb, ≥95% identity).

## Data Availability

BioSamples identify the specimen as SEPT_11B. In addition, GCF_053477285.1 is linked to PRJNA1332763 and SAMN51758283, and PRJNA1332763 now has linked to SRA records (view more, tab, click SRA SRS30377662): SRX34190764, SRX34190763, SRX34019630. https://www.ncbi.nlm.nih.gov/datasets/genome/GCF_053477285.1/ BioProject: PRJNA1332763, BioSample: SAMN51758283; Sample name: SEPT_11B “Crassostrea virginica isolate:SEPT_11B Genome sequence” is now coincided and linked with all raw data. The GitHub repository of all our lab developed scripts/codes are available on: https://github.com/jh-yale/Eastern-oyster-reference--genome
