# Supplementary material for: Chromosomal-Level Whole Genome Assemblies of Crassostrea Virginica
Source: bioRxiv. 2026 Oct 19:2026.10.13.758928. Preprint. [Version 1] doi: 10.64898/2026.10.13.758928 (PMC13738429; doi:10.64898/2026.10.13.758928)
Supplement: Supplementary file 1 [file media-1.pdf]

Supplementary Information (SI)

SI-01

Assembly workflow

A detailed workflow

The *C. virginica* reference genome was assembled *de novo*. The workflow contains 10 main stages.

Stage 1: Data Generation and Deposit to the Public Domain

Respective sequencing platforms are:

Revio, NovaSeqxPlus for PacBio HiFi, WGS, and Hi-C.

Deposit raw data generated for this project on the NCBI data repositories.

Stage 2: Data Quality Control

Assessing, cleaning tall raw data for high accuracy. Quality Trimming: We used tools FastQC to evaluate raw read quality. Raw reads were first assessed with FastQC v0.11.9 and Trimmomatic to trim low-quality bases and remove adapter sequences. Merqury was used to obtain proper QV scores. . The Merqury QV score is based on chromosome-by-chromosome analysis with separate WGS and PacBio datasets.

Stage 3: De Novo Assembly

Contig Construction: PacBio HiFi long reads were assembled de novo using Hifiasm to generate the primary contig assembly. Illumina WGS reads and Hi-C data were subsequently aligned to the assembly using tools including minimap2, BWA-MEM, and SAMtools for downstream polishing, validation, and scaffolding.

Stage 4: Scaffold Chromosome Assembly

Contigs with short pieces were separated by unresolved repetitive elements. Hi-C data were processed using Juicer and the 3D-DNA scaffolding pipeline to order, orient, and connect contigs into chromosome-scale scaffolds. The resulting scaffolds were then manually reviewed and finalized into the chromosome-level assembly.

Stage 5: Polishing and Gap-closure

Error Correction: we mapped short reads raw data back to the assembled scaffolds, using aligners Juicer and BWA-MEM. Then ran the polishing tool NextPolish to fix potential single nucleotide errors and short insertions and deletions.

Gap Closure: Assembly continuity was improved through PacBio HiFi read alignment, polishing with NextPolish, Hi-C–based scaffolding, and manual curation to resolve remaining gaps and assembly error.

Stage 6: Quality Assessments

To evaluate the structural and informational integrity of the assembled genome, we used QUAST to evaluate statistical completeness including contiguity metrics such as N50 (the length of the shortest contig in the set that contains 50% of the total genome length).

To assess biological completeness, we used BUSCO to capture the presence of known conserved orthologs and gene families in the assembly.

Stage 7: Genome Annotation

Repeat masking: We used Repeat Masker to identify and mask repetitive regions that can cause false gene predictions.

Gene annotation: BRAKER was used to integrate RNA-sequencing (transcriptome) data with ab initio gene prediction using AUGUSTUS to generate gene models and identify coding sequences.

Stage 8: Contamination assessment

Contamination was assessed using three complementary approaches. First, the assembled genome was evaluated using the NCBI Foreign Contamination Screen (FCS-GX). Second, GC content versus sequencing coverage analyses were performed independently using both Illumina WGS and PacBio HiFi reads. Third, we evaluated taxonomic classification using Kraken2.

.

Stage 9: Our lab developed scripts/codes are deposited on the GitHub repository:

https://github.com/jh-yale/Eastern-oyster-reference--genome

Note that scrips were written in Shell to run the original tools. Several tools required several Shell scripts and finalized by NextFlow programs.

Stage 10: The chromosome-level assembly is deposited in the NCBI Assembly database.

https://www.ncbi.nlm.nih.gov/datasets/genome/GCF_053477285.1/ .

SI-02

Seven Tables (Table S1-Table S7) and a PacBio document

Table S1. Chromosome-level summary of repeat content in the C. virginica assembly.

Breakdown of total bases and genomic proportions for major repeat classes (DNA transposons, LINEs, LTRs, SINEs, and unclassified elements) across each chromosome.

Chromosome	DNA Transposons(%)	LINE(%)	LTR(%)	SINE(%)	Unknown Repeats(%)	Total(%)
1	7.816	3.825	8.141	4.261	24.448	48.491
2	6.591	3.291	6.617	2.734	19.044	38.277
3	7.473	3.606	7.558	5.809	30.388	54.834
4	7.188	3.727	7.287	3.217	20.493	41.912
5	6.666	3.417	6.49	2.723	18.529	37.825
6	7.254	3.704	6.895	4.114	23.159	45.126
7	7.519	3.146	7.702	3.854	22.769	44.99
8	6.723	2.945	6.568	2.724	17.888	36.848
9	6.127	2.731	6.055	2.255	16.305	33.473
10	7.452	3.597	7.285	4.415	26.553	49.302
Total	7.091	3.415	7.086	3.621	21.974	43.187

Table S2. Telomere contents identified across the *C. virginica* genome.

Summary of putative telomeric repeat motifs, their copy numbers, and chromosomal locations detected at scaffold ends.

Chromosome	Start	End	Total Array Length(bp)	Period(bp)	Copy Number	Percent Matches
2	5,089	5,169	81	6	13.5	100
2	1,113	1,201	89	6	14.7	97
3	9,765	9,840	76	6	12.7	100
6	50,070,953	50,071,028	76	6	12.7	100
6	50,067,006	50,067,074	69	6	11.5	100
6	50,074,915	50,074,978	64	6	10.7	100
8	8,756	8,825	70	6	11.7	100
8	48,945,203	48,945,266	64	6	10.7	100
8	4	3,076	3073	6	512.2	99
8	4,304	4,848	545	6	90.7	99
9	48,405,959	48,406,033	75	6	12.5	100
10	37,759,184	37,759,680	497	6	82.8	100
10	37,755,233	37,755,308	76	6	12.7	100

Table S3. Putative centromeric satellite arrays identified across the *C. virginica* genome.

Coordinates, repeat unit lengths, copy numbers, and GC content of tandem satellite arrays consistent with centromeric signatures.

Chromosome	Start	End	Total Array Length(bp)	Period(bp)	Copy Number	Percent Matches
1	35,162,572	35,213,205	50,634	864	58.1	90
1	35,253,706	35,280,255	26,550	864	30.8	98
1	35,284,248	35,295,986	11,739	863	13.6	97
1	35,332,757	35,343,344	10,588	863	12.3	98
3	30,996,317	31,046,328	50,012	849	58.7	96
6	22,417,007	22,440,537	23,531	874	26.1	83
6	22,463,396	22,501,347	37,952	988	38.4	97
7	17,238,024	17,260,541	22,518	925	24.1	90
7	17,304,445	17,337,085	32,641	964	34.0	96
8	29,605,286	29,639,983	34,698	896	38.7	98
8	29,680,791	29,693,725	12,935	900	14.4	96
9	20,159,148	20,199,590	40,443	846	46.3	81
9	20,247,311	20,270,222	22,912	862	26.8	85
10	16,309,141	16,389,363	80,223	869	92.5	98
10	16,428,141	16,477,937	49,797	829	59.2	94

Table S4. Step-by-step metrics tracking across the assembly workflow.

Metric	Stage 1: Initial contigs	Stage 2: Post-deduplication	Stage 3: Post-scaffolding	Stage 4: post-manual haplotig removal
Total size (bp)	591927889	548049328	532076140	529025936
Number of scaffolds	222	120	10	10
Number of contigs	222	120	77	81
Contig N50 (bp)	29305747	32788167	16475059	16475059
Scaffold N50 (bp)			56120932	55461147
GC (%)	35.03	34.97	34.94	34.94
BUSCO complete - C (%)	99.8	99.7	99.7	99.7
BUSCO single-copy - S (%)	93.9	97.6	98.1	98.6
BUSCO duplicated - D (%)	5.9	2.1	1.6	1.1
BUSCO fragmented - F (%)	0.2	0.2	0.1	0.1
BUSCO missing - M (%)	0	0.1	0.2	0.2

Table S5. Program tools and corresponding scripts and outputs for assembly

Program tool	Script	Output
FastQC	HiC_QC.nf	FastQC html/zip
Trimmomatic	HiC_QC.nf	trimmed fastq
Hifiasm	assembly.nf	primary.fa
NextPolish	polish.nf	polished.fa
Purge_Dups	purge_dup.sh	dedup.fa
Juicer	HiC_prep_genome.sh	.hic/.mnd
3D-DNA	scaffolding.sh	scaffold.fa
QUAST	asm_eva.nf	quast report
BUSCO	asm_eva.nf	busco report

Table S6. All scripts and their functional purposes with input and output, Available on GitHub

https://github.com/jh-yale/Eastern-oyster-reference--genome

Script Name	Pipeline Step	Program(s) Used	Purpose / Function	Key Parameters & Settings	Input File(s)	Output File(s)	Notes
HiC_QC.nf	Hi-C Quality Control / Preprocessing	Nextflow, FastQC, Trimmomatic, fastp	Performs quality trimming and QC of paired-end Hi-C reads before scaffolding.	params.dir=/home/zl436/palmer_scratch/oyster_genome; params.reads=HiC/*_{1,2}.fastq.gz; Trimmomatic PE; -threads 5; ILLUMINACLIP:TruSeq3-PE.fa:2:30:10; HEADCROP:15; TRAILING:5; SLIDINGWINDOW:10:30; MINLEN:50; fastp --trim_poly_g; --trim_poly_x; --poly_x_min_len 10; -l 50; FastQC -t 4	Raw paired Hi-C FASTQ files: HiC/*_{1,2}.fastq.gz	Trimmed Hi-C reads: *_trimmed.fastq.gz; poly-G/poly-X cleaned reads: *_trimmed_grmv.fastq.gz; FastQC reports: *_fastqc.zip	Nextflow workflow for Hi-C read cleaning and QC. Uses containerized Trimmomatic, fastp, and FastQC.

HiC_prep_genome.sh	Hi-C Genome Preparation	Apptainer, Juicer v2.0.1, BWA, Java, Python	Prepares the chromosome-level genome assembly for Hi-C scaffolding by indexing the genome, generating restriction enzyme site positions for the Arima Hi-C library, and configuring the assembly for Juicer.	--cpus-per-task=12; --mem-per-cpu=5G; --time=40:00:00; apptainer pull docker://aidenlab/juicer:v2.0.1; bwa index; generate_site_positions.py Arima; juicer.sh --assembly -t 12	primary_dedup_chr_masked_hp_sealed.fa	final_Arima.txt; Arima_final.sizes; BWA index files; Juicer assembly preparation files	Prepares the genome for Hi-C scaffolding using the **Arima restriction enzyme** protocol. Generates the restriction site files and genome index required by Juicer before running the scaffolding pipeline.
braker_setup.sh	Gene Annotation Setup	Apptainer, BRAKER3, AUGUSTUS	Downloads the BRAKER3 container and prepares the AUGUSTUS configuration files needed for gene prediction.	--cpus-per-task=2; --mem-per-cpu=8G; --time=1:00:00; apptainer pull docker://teambraker/braker3:latest; --bind $PWD:/data	Docker image: teambraker/braker3:latest	braker3.sif; augustus_config/	Environment setup only; does not perform gene prediction itself.

gene_pred.sh	Gene Annotation	Apptainer, BRAKER3, AUGUSTUS, GeneMark-ETP	Runs BRAKER3 gene prediction on the soft-masked chromosome-level assembly using RNA-seq BAM evidence.	--cpus-per-task=16; --mem-per-cpu=5G; --time=23:00:00; apptainer pull docker://teambraker/braker3:latest; --genome=chr_asmbl_masked.fa; --bam=rna_merged.bam; --softmasking; --threads=16; --species=oyster; --gff3; --useexisting	chr_asmbl_masked.fa; rna_merged.bam; augustus_config/	gene_pred/; GFF3 gene annotation output from BRAKER/AUGUSTUS	Main gene prediction script; this one should likely be uploaded because it contains the actual BRAKER parameters, unlike braker_setup.sh, which only prepares the environment.
align_pacbio.sh	Assembly Validation / Read Alignment	Minimap2, SAMtools	Aligns PacBio HiFi reads to the final chromosome-level assembly and produces an indexed BAM file for downstream analyses (e.g., polishing, coverage analysis, validation).	--cpus-per-task=16; --mem=64G; --time=5:00:00; minimap2 -d (genome indexing); -ax map-hifi; -t ${SLURM_CPUS_PER_TASK}; samtools view; samtools sort; samtools index	chr_asmbl.fa; 11S_pb.fastq.gz	chr_asmbl_hifi.bam; chr_asmbl_hifi.bam.bai; chr_asmbl.fa.mmi	Uses the map-hifi preset specifically designed for PacBio HiFi reads. Produces the sorted and indexed alignment used for downstream analyses.

extract_protein.sh	Post-processing / Gene Annotation	Apptainer, gffread	Extracts predicted protein, CDS, and transcript sequences from the BRAKER GFF3 annotation using the final masked reference genome.	--cpus-per-task=2; --mem=8G; --time=01:00:00; apptainer pull docker://dceoy/gffread; -y proteins.fa; -x cds.fa; -w transcripts.fa	primary_dedup_chr_masked.fa ; braker.gff3	proteins.fa; cds.fa; transcripts.fa	Uses **gffread** to generate the FASTA files typically released with a genome annotation. The _hp version appears to perform the same task on a different assembly and is probably not needed for the reference genome repository.

annotate.nf	Gene Annotation / Assembly Evaluation	Nextflow, FastQC, fastp, QUAST, BUSCO, Meryl, Merqury, RepeatModeler, RepeatMasker, STAR, SAMtools, BRAKER3, StringTie, MUMmer4, AUGUSTUS	Large annotation/evaluation workflow that performs read QC/trimming, assembly evaluation, repeat masking, RNA-seq alignment, BAM merging, and BRAKER gene prediction.	params.threads=8; FastQC -t 8; fastp --trim_front1 15 --trim_front2 15 --cut_mean_quality 20 -l 50; QUAST -t 8; BUSCO -m genome -l mollusca_odb10 -c 8; Meryl k=21; Merqury merqury.sh; RepeatMasker -pa 8 -xsmall; STAR --runMode genomeGenerate, --outSAMtype BAM SortedByCoordinate; SAMtools merge -@ 8; BRAKER --softmasking --threads=16 --species=oyster	RNA FASTQs: /home/zl436/palmer_scratch/fastq/rna/*_{1,2}.fastq.gz; WGS FASTQs: WGS/*_{1,2}.fastq.gz; genome: primary_dedup_chr.fa; repeat library: custom_repeats_dedup.fa	FastQC reports; trimmed RNA reads; QUAST report; BUSCO report; Meryl k-mer DB; Merqury outputs; masked genome; STAR genome index; RNA BAMs; rna_merged.bam; braker_output/*	This is a broad workflow combining several steps. It overlaps with separate scripts like gene_pred.sh, but it is useful because it documents the full annotation/evaluation pipeline and exact software containers/parameters.

asm_eva.nf	Assembly Evaluation	Nextflow, FastQC, fastp, QUAST, BUSCO, Meryl, Merqury, RepeatModeler, RepeatMasker, STAR, SAMtools, BRAKER2, StringTie, MUMmer4, AUGUSTUS	Evaluates the final genome assembly by assessing contiguity (QUAST), gene completeness (BUSCO), k-mer quality (Merqury), repeat content, and supporting annotation analyses.	params.threads=8; QUAST -t 8; BUSCO -m genome -l mollusca_odb10; Meryl k=21; greater-than 3 k-mer filtering; Merqury merqury.sh; RepeatMasker -pa 8 -xsmall; BRAKER2 --softmasking --cores 8 --species=oyster; STAR --runMode genomeGenerate	WGS FASTQs (*_trimmed_grmv.fastq.gz); RNA FASTQs; primary_dedup_chr_masked_hp_sealed.fa; reference genome; repeat library	QUAST report; BUSCO report; filtered Meryl database; Merqury reports (QV/completeness); masked genome; STAR index; RNA BAMs; BRAKER2 outputs; MUMmer comparison files	This workflow is focused on **assembly evaluation**. Although it contains optional annotation processes, most of those are commented out. The active workflow primarily generates the quality metrics (QUAST, BUSCO, Merqury) that were ultimately used to assess the final reference genome.
assembly.nf	Genome Assembly	Nextflow, Hifiasm v0.24.0	Performs the **de novo assembly** of the oyster genome from PacBio HiFi reads using Hifiasm. This is the initial genome assembly step of the reference genome pipeline.	hifiasm v0.24.0; -o contigs; -t 16 (16 CPU threads); --cpus=16	PacBio/hifi_reads/11S_pb.fastq.gz	contigs* (primary assembly contigs produced by Hifiasm)	Core assembly workflow. Uses PacBio HiFi reads as input to generate the initial contig assembly that is later polished, purged, scaffolded, and annotated.

generate_hic_file.sh	Hi-C Processing / Hi-C Contact Map Generation	Java, Juicer Tools v2.20.00, Apptainer	Generates the final **.hic contact map** from Juicer alignment results for visualization and downstream Hi-C scaffolding/analysis.	--cpus-per-task=4; --mem-per-cpu=16G; --time=12:00:00; juicer_tools pre; --threads 4; -q 30 (minimum mapping quality); resolutions 5000,10000,25000,50000,100000 bp	aligned/merged_nodups.txt; Arima_final.sizes; primary_dedup_chr_masked_hp_sealed.fa (prepared genome)	primary_dedup_chr_masked_hp_sealed.hic	Converts Juicer alignment output into the binary **.hic** contact matrix used by **Juicebox** and other Hi-C visualization/scaffolding tools. The mapping quality threshold (-q 30) retains high-confidence Hi-C contacts.

kmer_analysis.sh	Assembly Evaluation (k-mer Analysis)	Apptainer, Meryl v1.4.1, Merqury v1.4.1	Evaluates the quality and completeness of the assembled genome using k-mer analysis. Uses precomputed k-mer databases from HiFi and WGS reads to calculate Merqury quality metrics (QV, completeness, spectra plots).	--cpus-per-task=8; --mem-per-cpu=10G; --time=3:00:00; merqury.sh; Meryl k=21 (commented commands); evaluates using both **HiFi** and **WGS** k-mer databases	primary_dedup_chr.fa; hifi_kmer.meryl; wgs_kmer.meryl	merqury_draft2_hifi*; merqury_draft2_wgs* (QV scores, completeness statistics, spectra plots)	Generates the **Merqury quality metrics** (including **QV score** and completeness) used to assess the final reference genome. The commented Meryl commands document how the k-mer databases can be generated, while the active commands run the final Merqury evaluation using existing databases.

polish.nf	Polishing / Assembly Evaluation	Nextflow, gfatools, QUAST, BUSCO, Meryl, Merqury, BWA, SAMtools, NextPolish	Converts Hifiasm GFA assemblies to FASTA, evaluates contigs, builds k-mer databases, runs Merqury, maps Illumina reads, and polishes the primary assembly using NextPolish with HiFi and Illumina reads.	Hifiasm GFA converted with gfatools gfa2fa; QUAST -t 8; BUSCO -m genome -l mollusca_odb10 -c 8; Meryl k=21; BWA mem -t 4; SAMtools filter -q 30; NextPolish task=best, rerun=3, parallel_jobs=6, multithread_jobs=5, SGS -max_depth 100 -bwa, HiFi -min_read_len 1k -max_depth 100, minimap2 -x map-pb	Hifiasm GFA files; primary.fa; PacBio HiFi reads 11S_pb.fastq.gz; trimmed WGS reads *{1,2}_trimmed_grmv.fastq.gz	FASTA assemblies; QUAST reports; BUSCO reports; Meryl k-mer DBs; Merqury outputs; sorted/filtered BAMs; genome.nextpolish.fasta	Main polishing workflow. Also includes early assembly evaluation steps for hap1, hap2, and primary Hifiasm assemblies before polishing the primary contig assembly.

post_review.sh	Hi-C Scaffolding / Post-review Assembly	Apptainer, 3D-DNA	Applies manual corrections from Juicebox/3D-DNA review to generate the final scaffolded genome assembly after Hi-C curation.	--cpus-per-task=8; --mem-per-cpu=10G; --time=48:00:00; apptainer pull docker://aakashsur/3d-dna; run-asm-pipeline-post-review.sh; --sort-output; -r primary_dedup.rawchrom.review.assembly	primary_dedup.fa; merged_nodups.txt; primary_dedup.rawchrom.review.assembly	Final post-review scaffolded assembly files generated by the 3D-DNA pipeline	Finalizes the chromosome assembly by incorporating **manual Hi-C corrections** made in Juicebox. This is the post-review step following 3D-DNA scaffolding and is a key part of producing the final reference genome.

purge_dup.sh	Haplotig Purging / Redundancy Removal	Minimap2, Purge_Dups, SAMtools	Identifies and removes redundant haplotigs from the primary assembly using PacBio HiFi read coverage and self-alignment, producing a deduplicated genome assembly.	--cpus-per-task=8; --mem-per-cpu=8G; --time=23:00:00; minimap2 -x asm20 (HiFi-to-assembly alignment); pbcstat; calcuts; split_fa; minimap2 -x asm5 -DP (self-alignment); purge_dups -2 -T <cutoffs> -c PB.base.cov; get_seqs -e	primary.fa; 11S_pb.fastq.gz	primary_dedup.fa; *.paf.gz; PB.stat; *.cutoffs; *.dups.bed; Purge_Dups log files	Core redundancy-removal step of the reference genome pipeline. Uses HiFi read coverage together with self-alignment to identify duplicated haplotigs before Hi-C scaffolding. This is the **Purge_Dups** step referenced in the manuscript.

repeat_masking.sh	Repeat Identification & Repeat Masking	Apptainer, RepeatModeler, RepeatMasker (Dfam TE Tools)	Builds a custom repeat database from the assembled genome using RepeatModeler, then masks repetitive elements in the genome with RepeatMasker to prepare the assembly for downstream gene annotation.	--cpus-per-task=16; --mem-per-cpu=8G; --time=120:00:00; BuildDatabase; RepeatModeler -threads 16 -LTRStruct; RepeatMasker -pa 16 -lib <custom_library> -xsmall	primary_dedup_chr_masked_hp_sealed.fa	final_genome_db; ${DBNAME}-families.fa (custom repeat library); soft-masked genome (*.fa.masked)	Generates a **species-specific repeat library** and performs **soft masking** of repetitive DNA. The -LTRStruct option improves detection of long terminal repeat (LTR) retrotransposons, and the resulting masked genome is used as input for BRAKER gene prediction.

scaffolding.sh	Hi-C Scaffolding	Apptainer, 3D-DNA, Java	Performs **Hi-C–based scaffolding** of the deduplicated genome assembly using the 3D-DNA pipeline and Hi-C alignment data to order and orient contigs into chromosome-scale scaffolds.	--cpus-per-task=12; --mem-per-cpu=8G; --time=48:00:00; apptainer pull docker://aakashsur/3d-dna; 3d-dna -r 0	primary_dedup.fa; merged_nodups.txt	Initial scaffolded assembly (*.assembly, scaffolded FASTA, and other 3D-DNA output files)	Core Hi-C scaffolding step of the reference genome pipeline. The -r 0 option disables automatic misjoin correction, allowing manual review and correction later in **Juicebox** before the final **post_review.sh** step.
filter_fragments.sh	Final Assembly Filtering	SeqKit	Filters the 3D-DNA scaffolded assembly to keep only large chromosome-scale scaffolds.	seqkit seq -m 20000000	primary_dedup.FINAL.fasta	primary_dedup_chr.fa	Keeps scaffolds ≥20 Mb. This likely produced the chromosome-level assembly file used for later evaluation and annotation.

calculate_depth.sh	Assembly Validation / Coverage Analysis	SAMtools, Python (bin_depth.py)	Calculates whole-genome sequencing depth from aligned reads and summarizes coverage into fixed-size genomic bins for assembly quality assessment.	samtools depth -d 10000; bin_depth.py -b 100000; --mem=40G; --time=2:00:00	11S_st_dr_filtered.bam	wgs_alignment_depth.txt; wgs_depth_binned.txt	Uses samtools depth to compute per-base coverage and bins coverage into 100 kb windows for downstream QC and coverage analysis.
bin_depth.py	Assembly Validation / Coverage Analysis	Python, pandas	Calculates the mean sequencing depth within fixed-size genomic bins from per-base coverage data generated by samtools depth.	-i input depth file; -o output file; -b bin size (default 10,000 bp; used with **100,000 bp** in calculate_depth.sh)	samtools depth output (wgs_alignment_depth.txt)	wgs_depth_binned.txt	Groups per-base coverage by genomic bins and computes the average depth for each bin. Used by calculate_depth.sh to summarize WGS coverage across the assembly.

summarize_depth.sh	Assembly Validation / Coverage Analysis	Bash, AWK	Summarizes WGS depth by scaffold, including mean, median, min, max, covered bases, and depth distribution.	Uses AWK arrays; calculates per-scaffold summary statistics; outputs scaffold-level depth distribution.	wgs_depth.txt	depth_summary_by_scaffold.tsv; depth_distribution_by_scaffold.tsv	Supports assembly validation by summarizing read-depth coverage across scaffolds. Useful for documenting coverage uniformity and potential problematic regions.
gff2bed.sh	Annotation Formatting	Bash, AWK, sort, cut	Converts BRAKER GFF3 gene annotations into a simplified BED-style file for downstream analyses.	Filters gene features; removes HiC_scaffold_ prefix; sorts by chromosome and start position	gene_pred/braker.gff3	CV_final.bed	Oyster-specific annotation formatting script. Uses final BRAKER annotation from the reference genome pipeline.
calculate_GC.sh	Assembly Validation / GC Content Analysis	BEDTools, SAMtools, AWK	Calculates GC content across the final genome assembly in fixed-size windows.	BIN_SIZE=200000; samtools faidx; bedtools makewindows; bedtools nuc	primary_dedup_chr_masked_hp_sealed.fa	gc.tsv; bins_200000.bed; bins_200000_nuc.tsv	Oyster-specific QC script. Computes GC content in 200 kb windows across the final assembly.

repeatmasker_out2bed.sh	Repeat Annotation	Bash, AWK	Converts RepeatMasker output into BED format for downstream genomic analyses and visualization.	Skips RepeatMasker header; converts coordinates to 0-based BED format; records repeat name and strand.	primary_dedup_chr_masked_hp_sealed.fa.out	all_repeats.bed	Post-processing utility for RepeatMasker output. Produces a BED file suitable for genome browsers and downstream analyses.
extract_unique_protein.sh	Gene Annotation Post-processing	Bash, AWK, sort	Extracts the longest protein isoform for each predicted gene and creates a non-redundant protein FASTA for downstream analyses.	Uses AWK to identify the longest isoform per gene; sorts gene IDs with sort -k1,1V.	proteins.fa	CV_final_protein_unique.fa	Post-processing step after extract_protein.sh. Produces a representative protein set for downstream analyses.

Table S7. Sequencing statistics table should be added, including sequencing depth, read counts/yield, read N50, and platform information for PacBio HiFi, Illumina WGS, and Hi-C datasets.

	Sequencing depth	Read counts	Read N50	Platform
PacBio HiFi	118.46×	10,877,578 reads	7,405	Revio
Illumina WGS	30.81×	88,515,995 read pairs	151	NovaSeq x Plus
Hi-C	N/A	330,467,201read pairs	151	NovaSeq x plus

Note: Sequencing depth values represent alignment depth across the final assembly.

Report for m84189_250113_210047_s1

Dataset details

Name: SEPT_11B

Path: /gpfs/gibbs/pi/ycga/pacbio/data/r84189_20250113_205244/1_A01/pb_formats/m84189_250113_210047_s1.hifi_reads.consensusreadset.xml

Unique ID: 74d69606-4f18-4506-a8d9-a91e7d12df8d

Created at: 2025-01-15T14:54:26.347Z

HiFi sequences: 10,877,578

HiFi bases: 68,977,516,064

Bio sample name: SEPT_11B

Well sample name: SEPT_11B

Run name: Run 01.13.2025 15:25

Movie name: m84189_250113_210047_s1

Instrument name: Revioli

ICS version: 13.3.0.253824

Number of child datasets: 0

Number of HiFi BAM files: 1

CCS Analysis Report

Summary Metrics

**HiFi reads**	10.9 M
**HiFi reads yield**	68.83 Gb
**HiFi reads length (mean)**	6.33 kb
**HiFi reads length (median, bp)**	5,299
**HiFi Read Length N50 (bp)**	7,405
**HiFi Read Quality (median)**	Q40
**HiFi Read Quality (median)**	40
**Base Quality ≥Q30 (%)**	96.18%
**HiFi Number of Passes (mean)**	22
**Missing adapters (%)**	19.01%

HiFi Read Length Summary

Read Length (kb)	Reads	Reads (%)	Yield (Gb)	Yield (%)
≥ 0	10,877,578	100	68.83 Gb	100
≥ 5,000	5,871,691	54	51.00 Gb	74
≥ 10,000	1,560,603	14	20.85 Gb	30
≥ 15,000	357,565	3	6.46 Gb	9
≥ 20,000	70,181	1	1.61 Gb	2
≥ 25,000	12,667	0	0.35 Gb	1
≥ 30,000	2,241	0	0.07 Gb	0
≥ 35,000	408	0	0.02 Gb	0
≥ 40,000	77	0	0.00 Gb	0

HiFi Read Quality Summary

Read Quality (Phred)	Reads	Reads (%)	Yield (Gb)	Yield (%)
≥ Q20	10,877,578	100	68.83 Gb	100
≥ Q30	8,478,295	78	51.26 Gb	74
≥ Q40	5,391,947	50	28.58 Gb	42
≥ Q50	2,955,974	27	13.32 Gb	19

Read Length Distribution: HiFi read length distribution

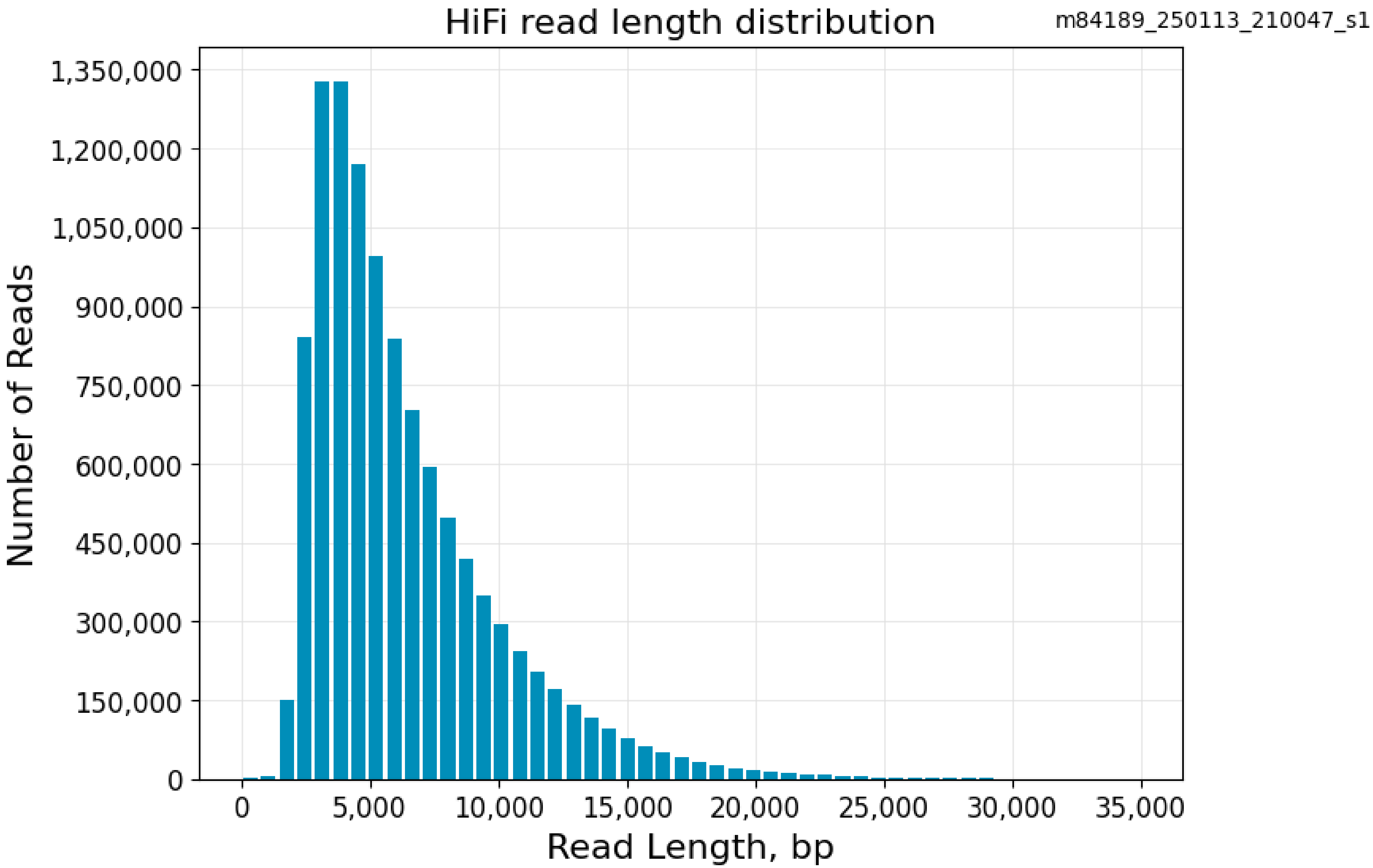

Read Length Distribution: Yield by HiFi Read Length

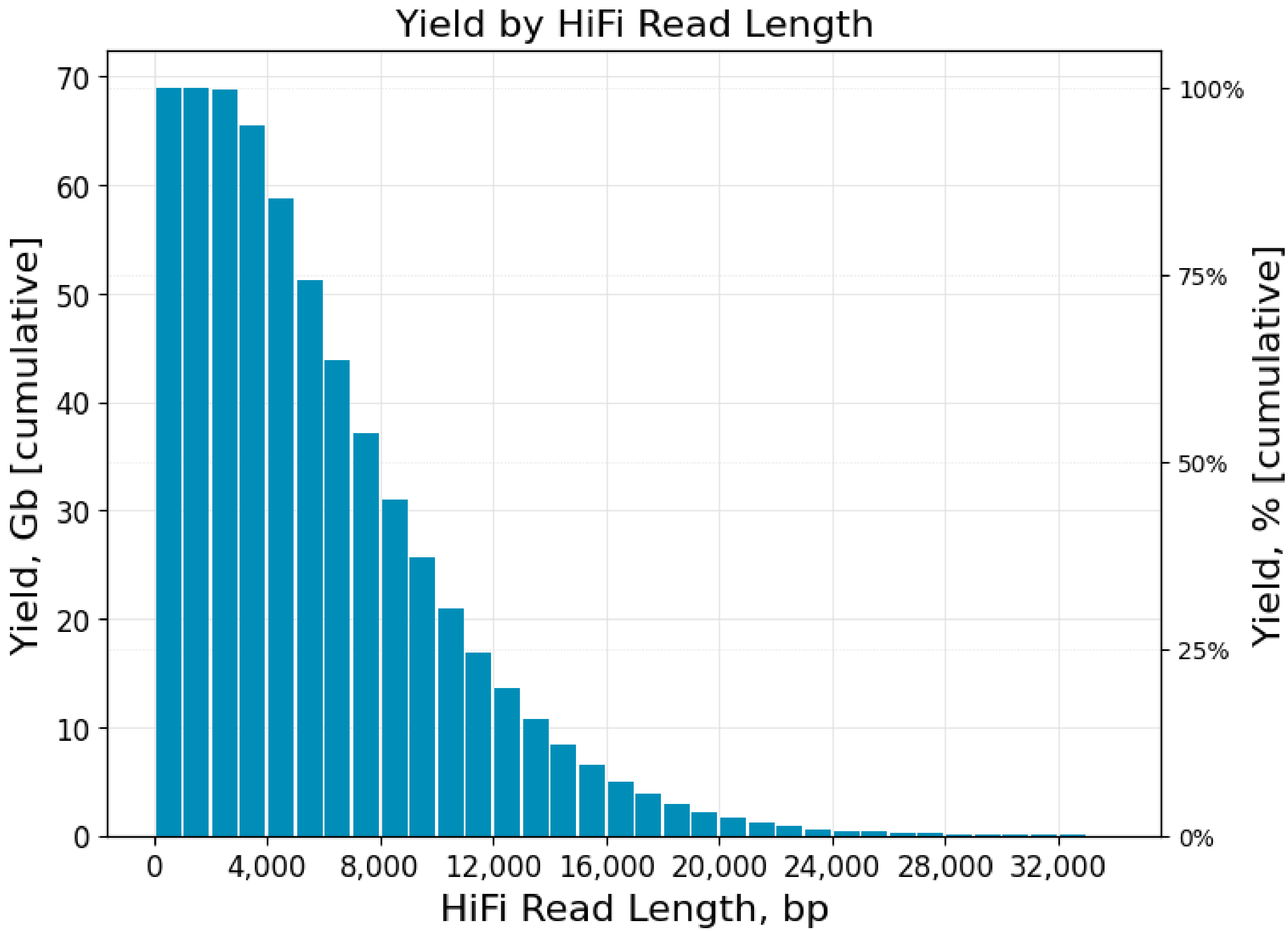

Read Length Distribution: Read length distribution

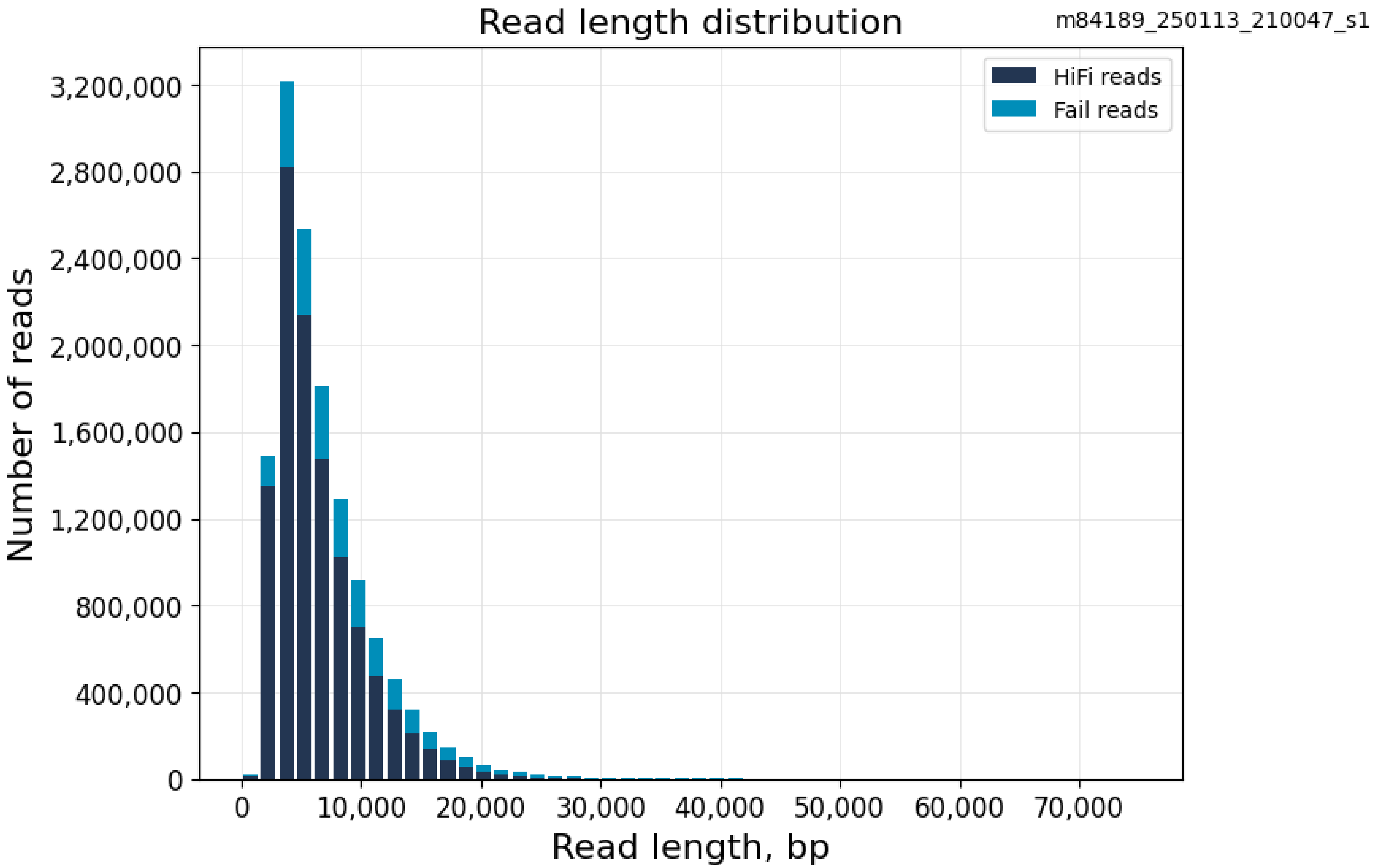

Number of Passes

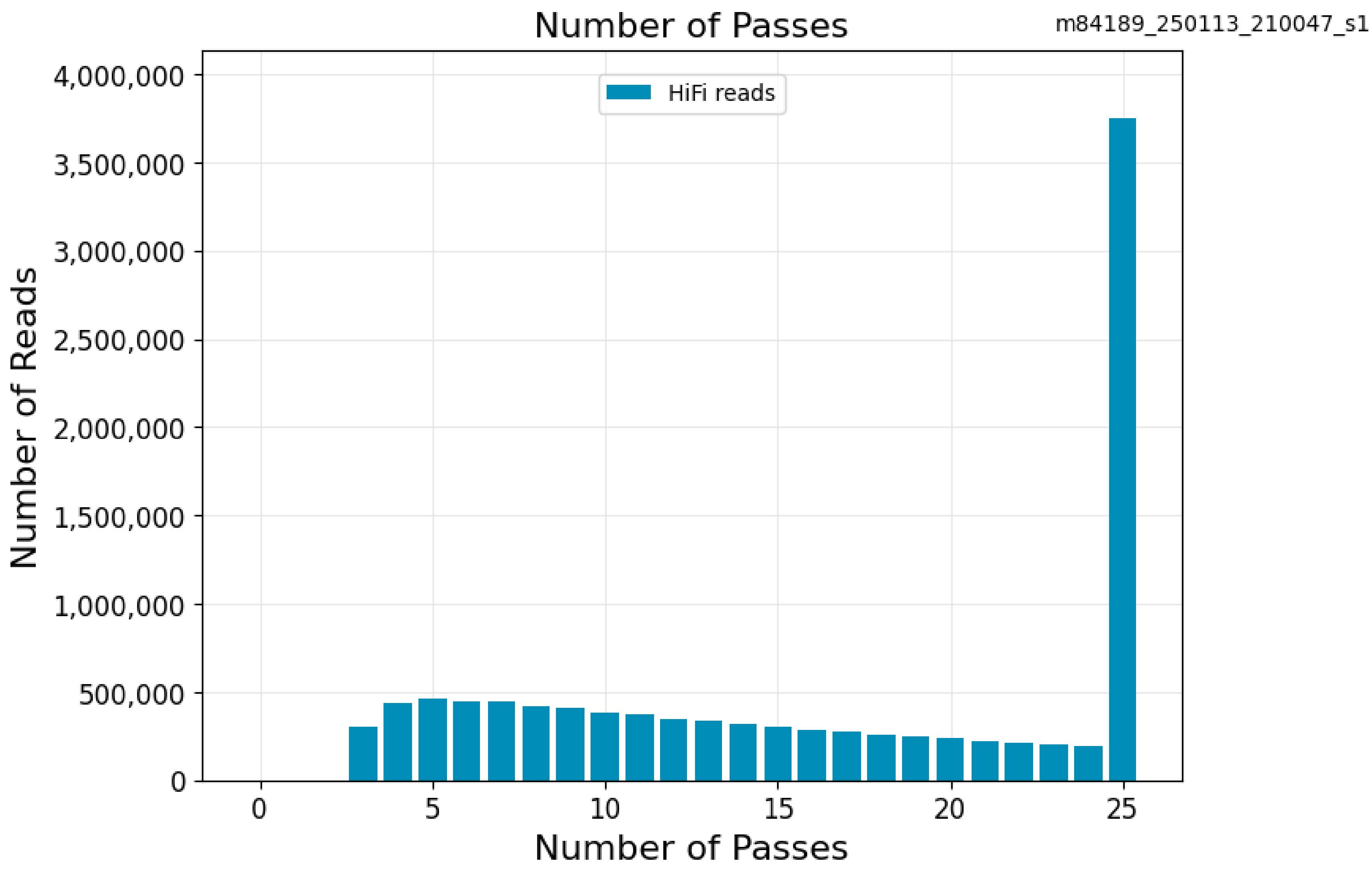

Read Quality Distribution

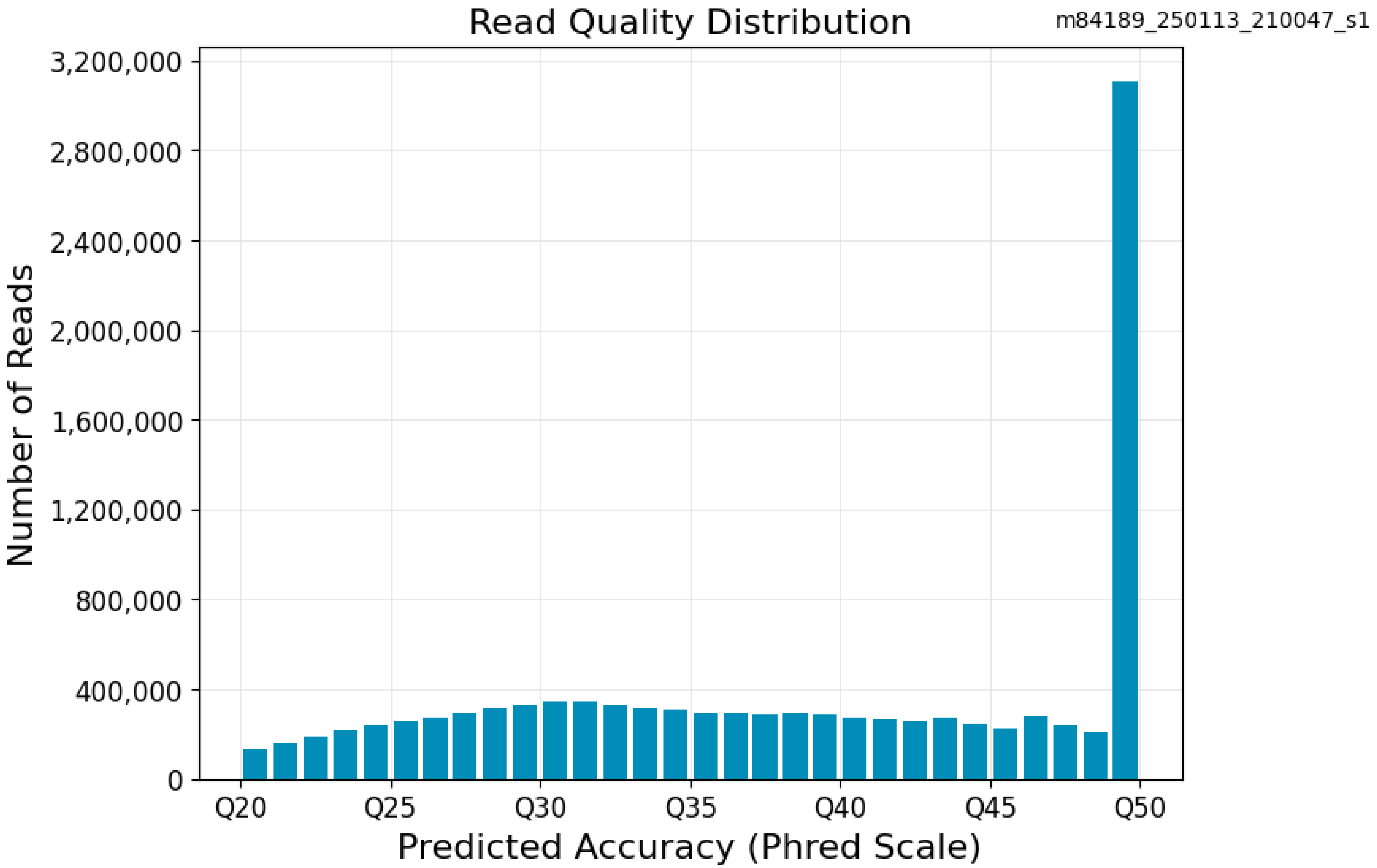

Predicted Accuracy vs. Read Length

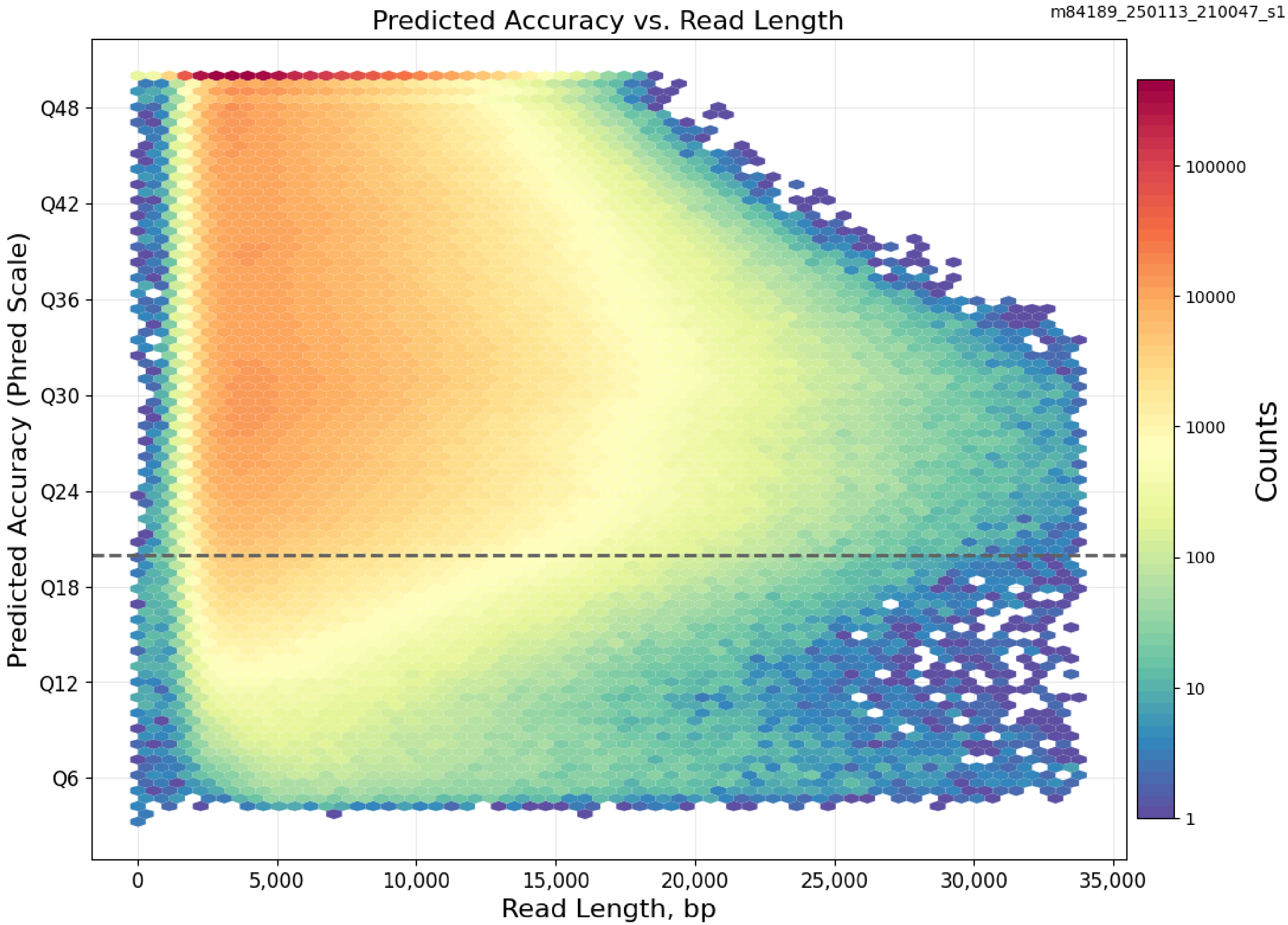

Methylation

Summary

Modification	Motif	Scored sites	Modified sites (Pr > 0.5)
5mC	CpG	97.4%	16.0%
6mA	A	0.3%	0.3%

Score distributions: 5mC at CpG

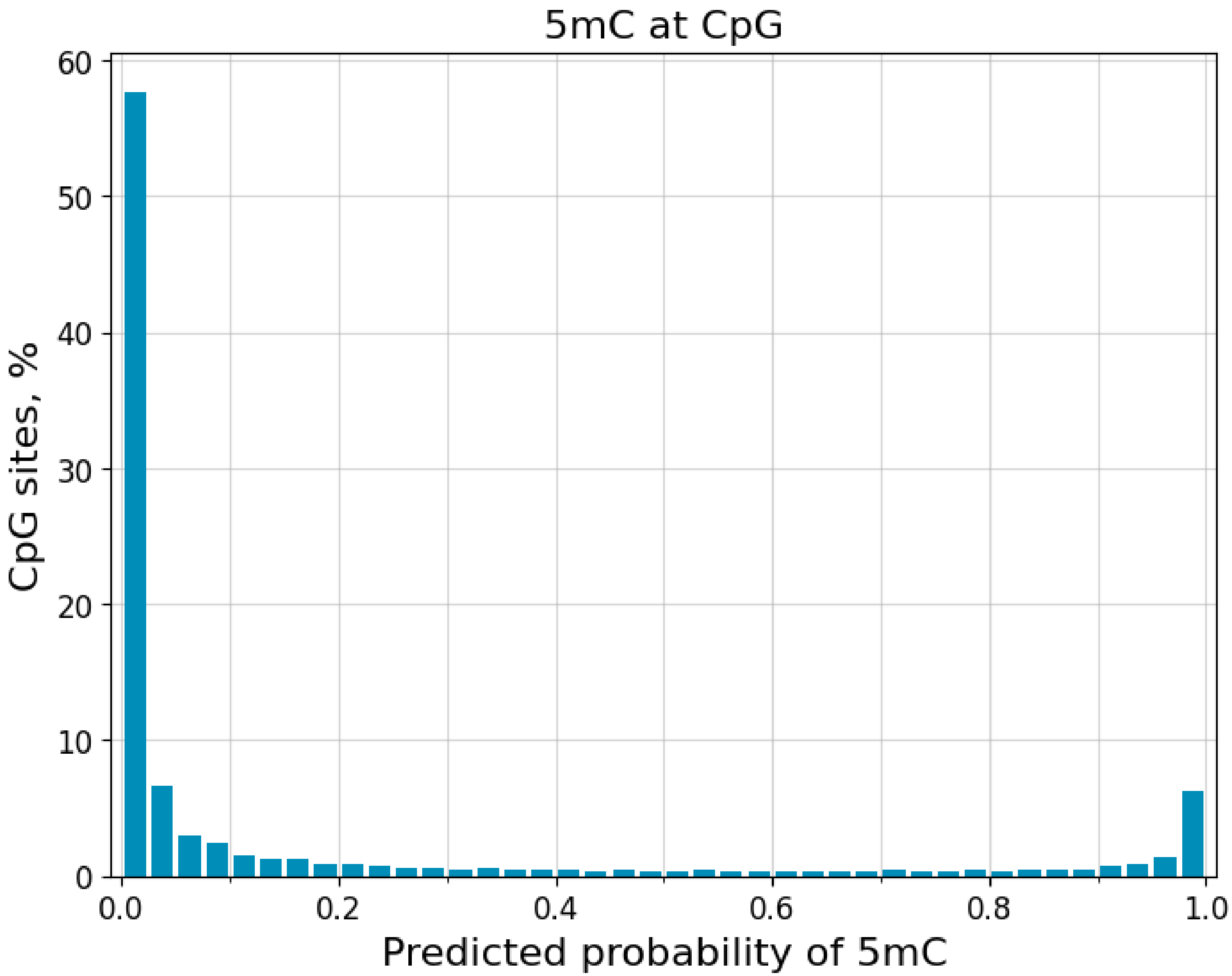

Score distributions: 6mA

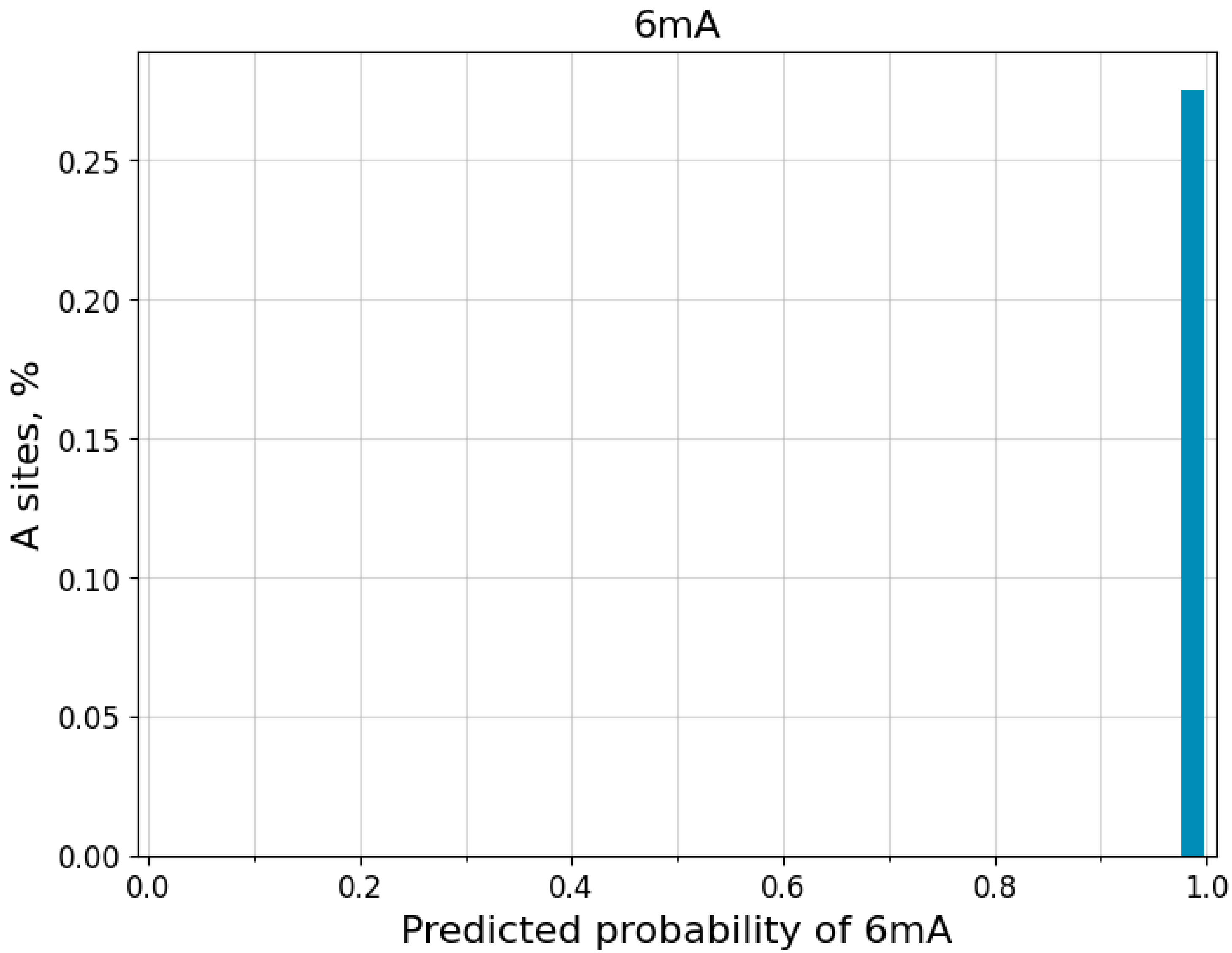

Loading Report

Summary Metrics

**Productive ZMWs**	25,165,824
**Productivity 0**	5,453,913
**Productivity 1**	19,542,790
**Productivity 2**	169,121

Raw Data Report

Summary Metrics

**Polymerase Read Bases**	1,555,639,150,564
**Polymerase Reads**	19,540,996
**Polymerase Read Length (mean)**	79.61 kb
**Polymerase read length (N50)**	153.25 kb
**Polymerase read length longest subread length (mean)**	10.53 kb
**Polymerase read length longest subread length (N50)**	15.75 kb
**Unique Molecular Yield**	171,983,568,896
**Local Base Rate**	2.15

Polymerase Read Length

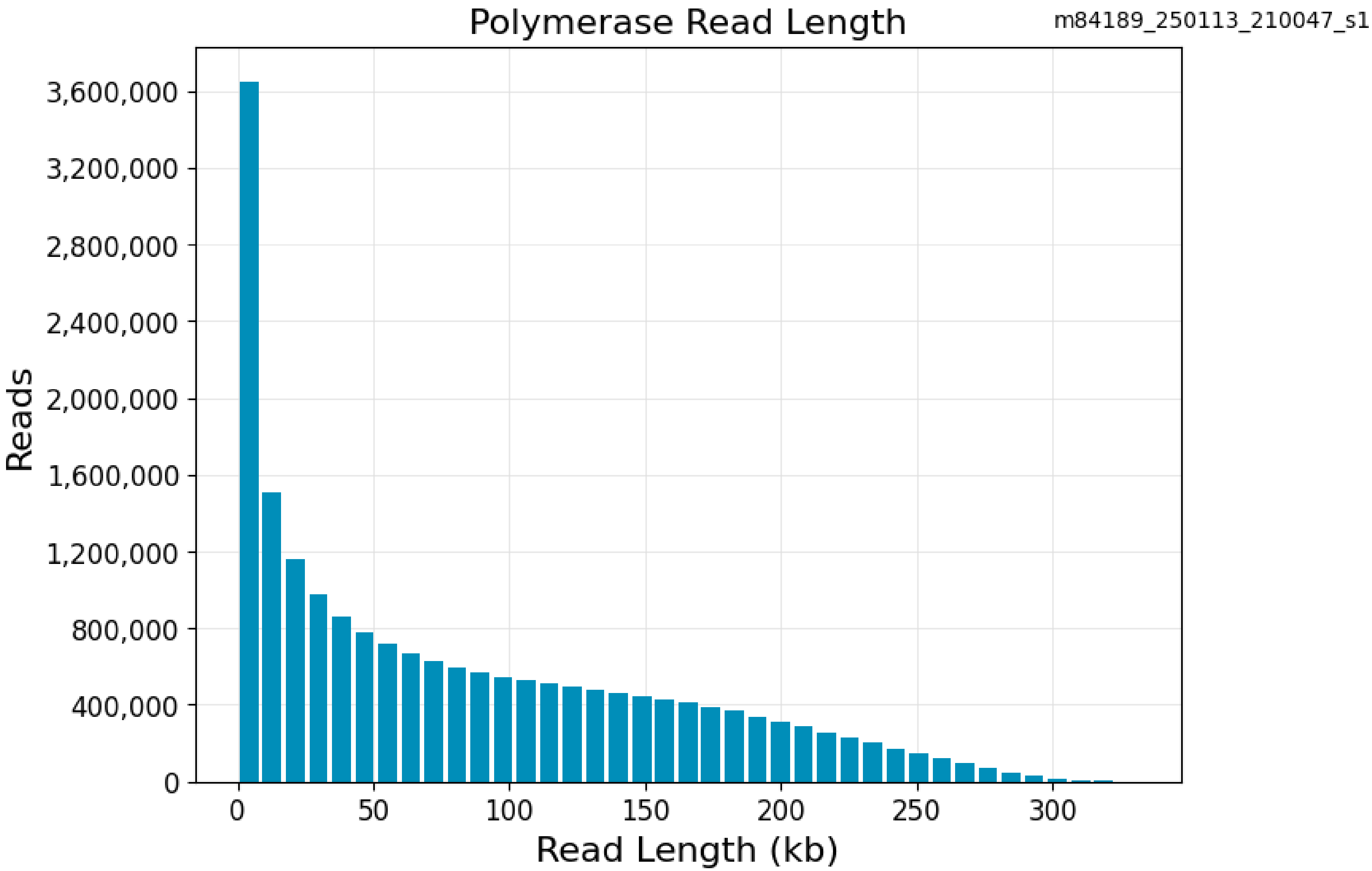

Longest Subread Length Versus Polymerase Read Length

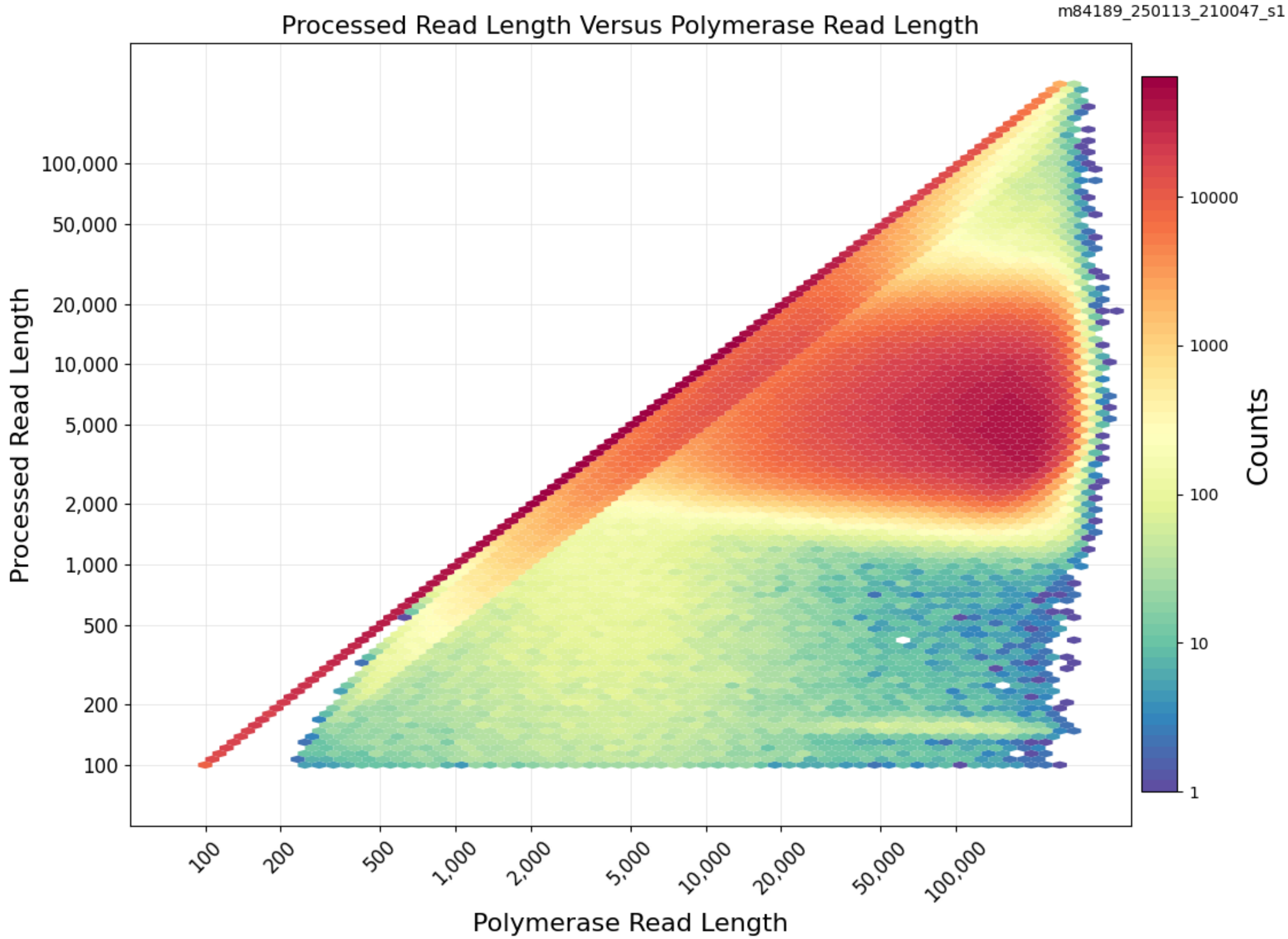

Base Yield Density

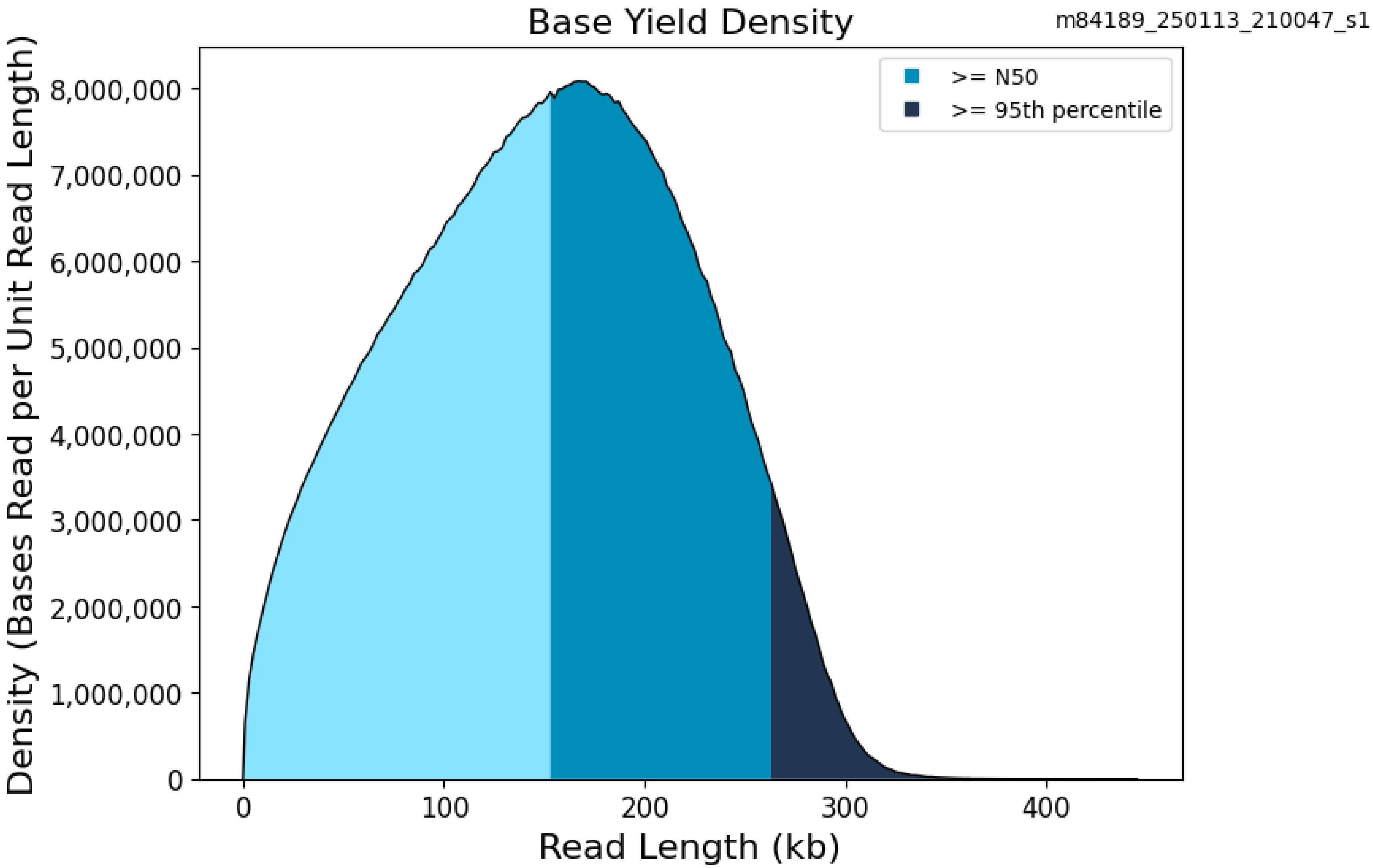

Control Report

Summary Metrics

**Number of Control Reads**	1,794
**Control Read Length Mean**	81,907
**Control Read Concordance Mean**	0.90
**Control Read Concordance Mode**	0.91

Control Read Length: Control Polymerase RL

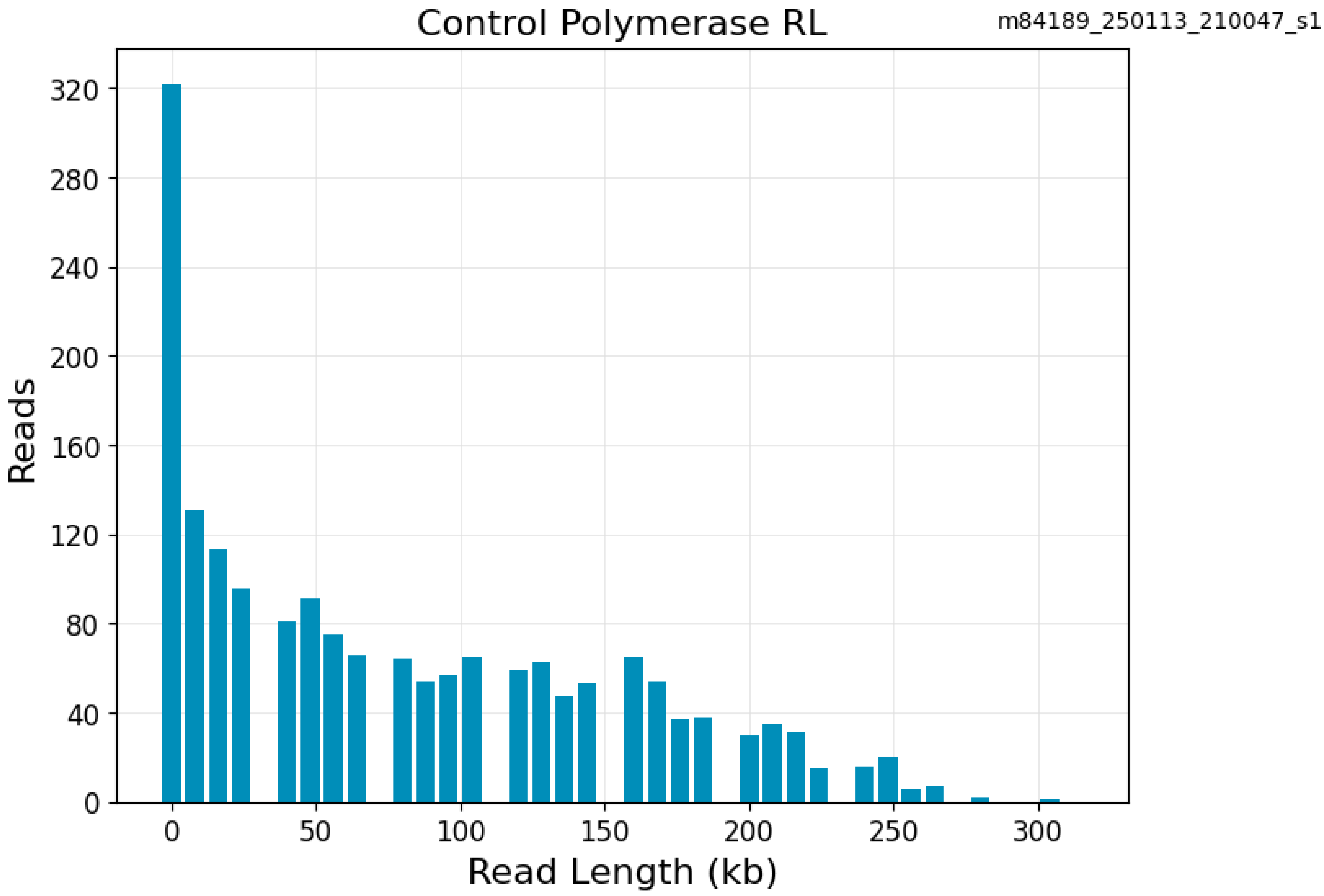

Control Read Quality: Control Concordance

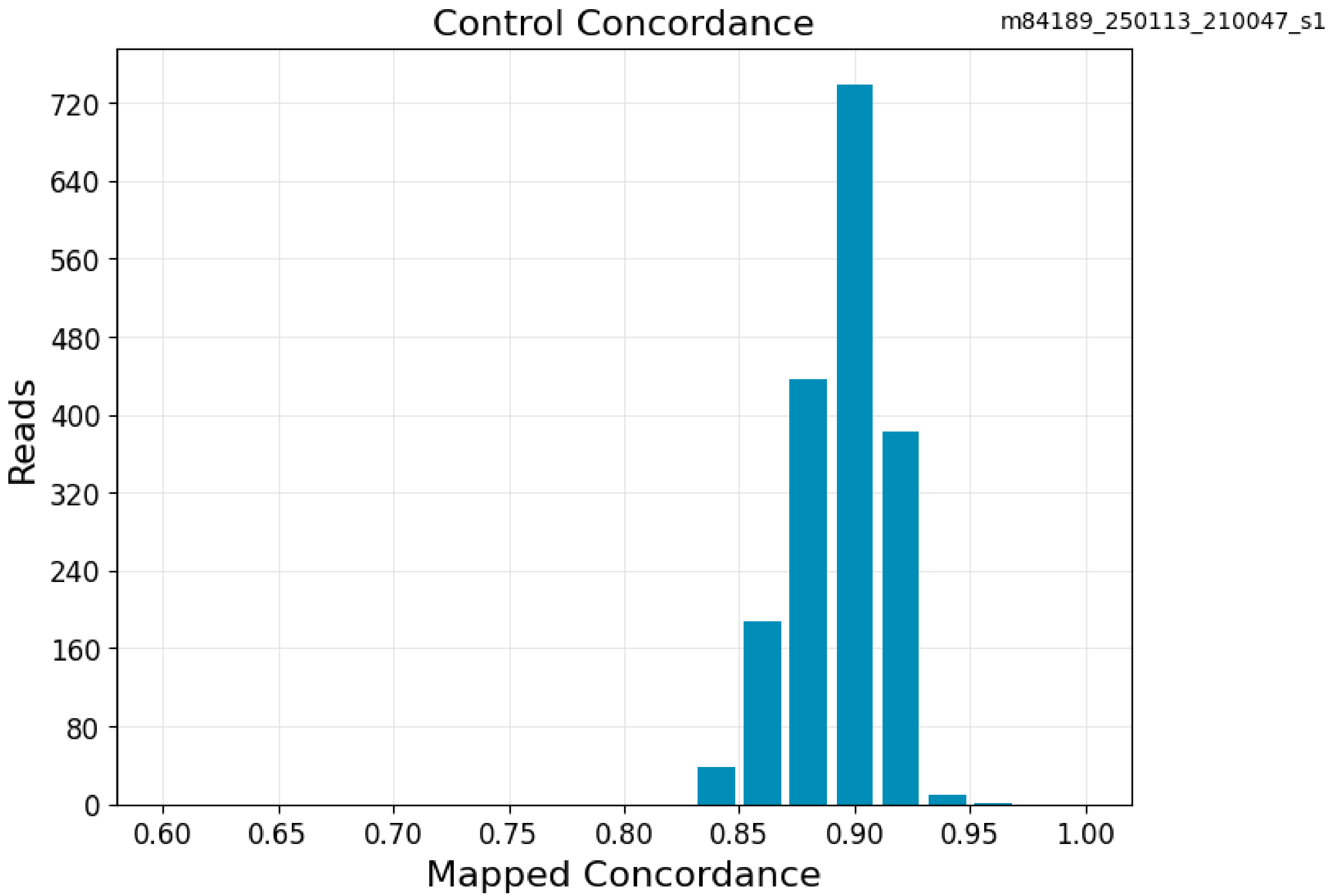

SI – 03

Contamination assessments in detail

Method overview

- Three complementary approaches

Index	Approach	Tool	Input
1	NCBI FCS-GX screen	NCBI automated pipeline	Assembled genome
2	GC vs coverage analysis	BWA + bedtools + R	Assembly + WGS reads (11S)
	GC vs coverage analysis	Minimap2 + bedtools + R	Assembly + PacBio reads (11S)
3	Taxonomic classification	Kraken2 (PlusPF-16 database)	Assembly, WGS, PacBio

NCBI FCS-GX

- Foreign Contamination Screening (FCS)
- NCBI mandatory contamination screen for genome assembly
- Reference genome published on NCBI -> It passed screening
- https://usegalaxy.eu/root?tool_id=ncbi_fcs_gx
- Results: zero sequences flagged for removal

FCS-GX results	Segment	Total bp	% genome
primary-div: molluscs	85	528,994,917	99.994%
inconclusive (within Animalia)	2	20,027	0.004%
low-coverage	1	8,879	<0.001%
same-kingdom-chimeric	1	2,113	<0.001%
**Sequences to remove**	**0**	**0**	**0%**

GC-Coverage Analysis

Rationale:

- Every organism has a characteristic GC content
- Scaffolds from contamination may have different GC content and low coverage

Workflow:

- Divide reference assembly into non-overlapping 100 kb windows.
- Calculate GC content of each window from the assembly sequence.
- Align WGS reads to the reference assembly (only primary alignments).
- Calculate mean read coverage for each window from the aligned reads.
- Plot GC content and coverage for all windows.
- Same for PacBio reads

GC-Coverage Analysis (WGS)

- Each dot represents one 100 kb window (5,294 windows).
- Single tight cluster at 35% GC, **~47× coverage**

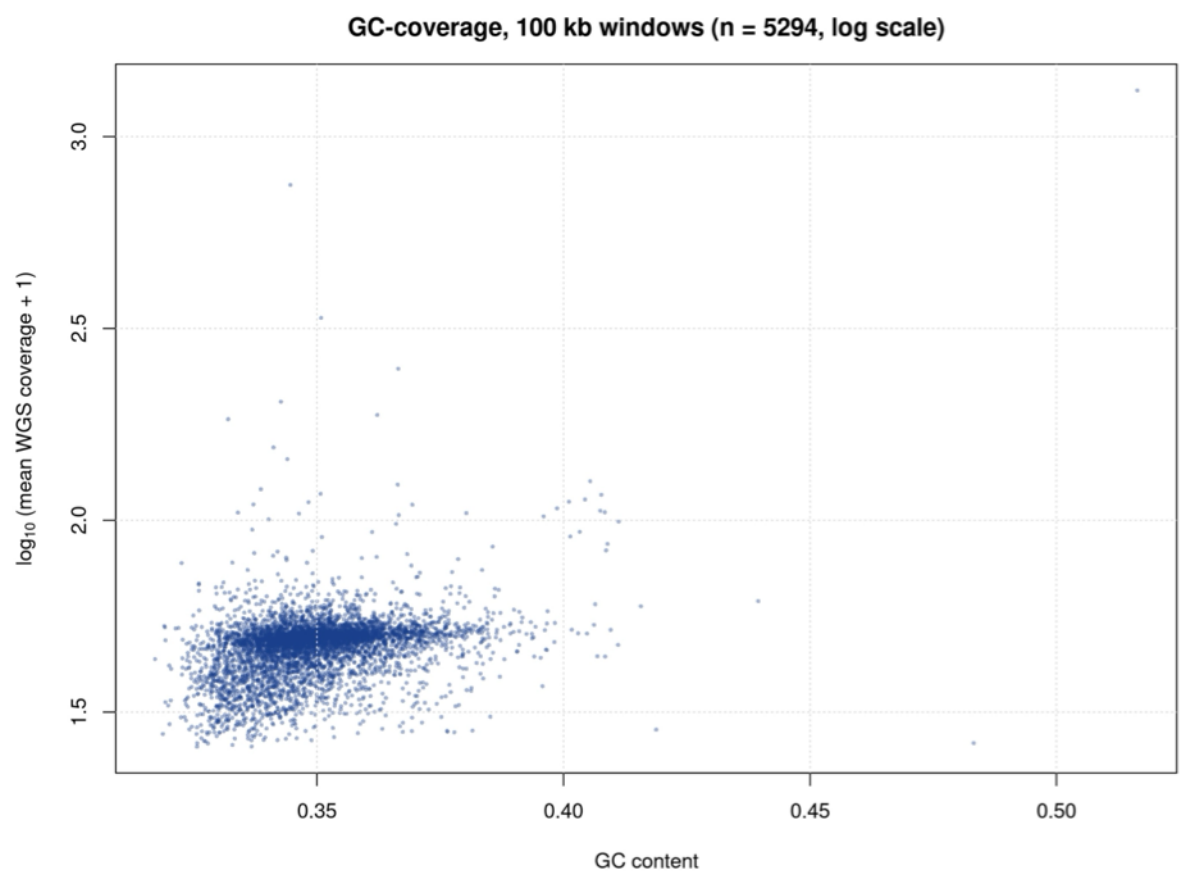

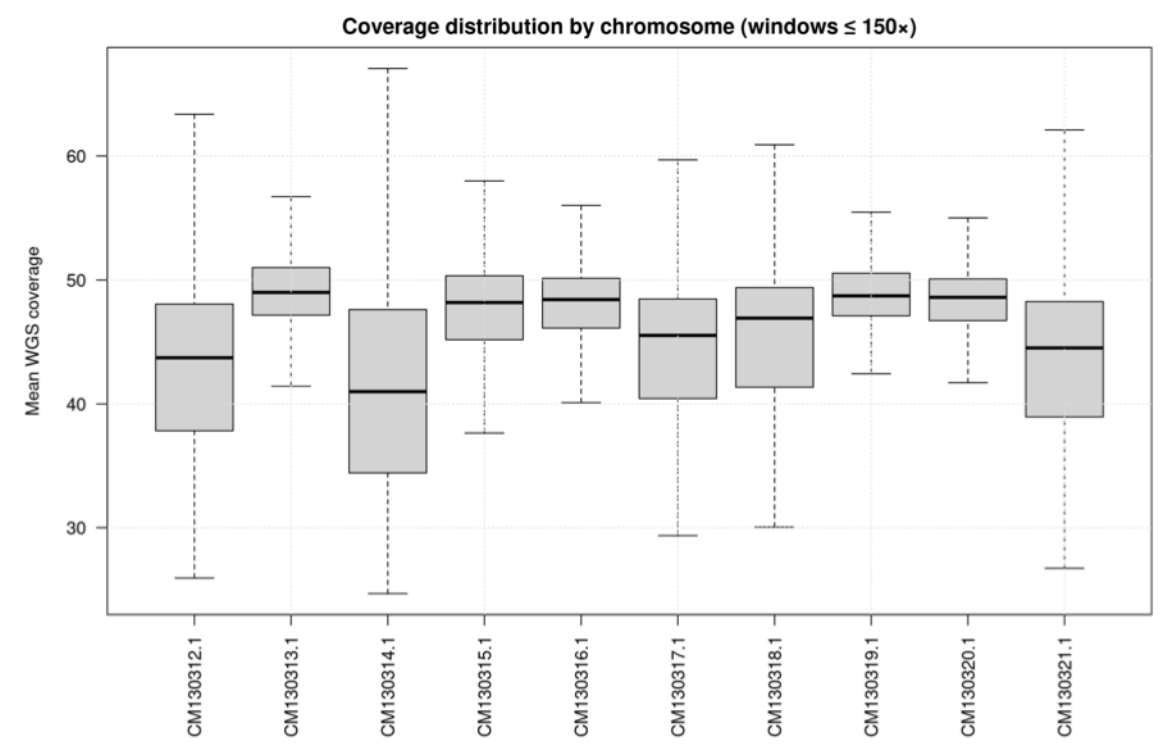

Note: Mean coverage of ~47×, calculated from primary BWA-MEM alignments without duplicate removal or MAPQ filtering in 100-kb windows for GC–coverage-based contamination assessment .

GC-Coverage Analysis (WGS)

- One outlier with high GC content and coverage
- Blast results: ribosomal RNA gene

Description	Scientific Name	Max Score	Total Score	Query Cover	E value	Per. Ident	Acc. Len	Accession
Crassostrea rhizophorae genome assembly, chromosome: 3	Crassostrea rhi...	13653	8.398e+05	71%	0.0	98.45%	56170271	OZ350604.1
PREDICTED: Crassostrea virginica large subunit ribosomal RNA (LOC144627123), rRNA	Crassostrea vir...	6970	17755	16%	0.0	100.00%	3774	XR_013565516.1
Crassostrea virginica 28S ribosomal RNA gene, partial sequence	Crassostrea vir...	6314	16293	15%	0.0	99.62%	3466	AY145400.1
Magallana gigas strain QD chromosome 10	Magallana gigas	6277	1.509e+05	28%	0.0	96.44%	38615286	CP048848.1
Magallana gigas genome assembly, chromosome: 10	Magallana gigas	6277	1.193e+05	22%	0.0	96.44%	37310742	OY970754.1

- rDNA clusters are multi-copy. Not foreign sequence

GC-Coverage Analysis (PacBio)

- Each dot represents one 100 kb window (5294 windows).
- Single tight cluster at 35% GC, **~114× coverage**

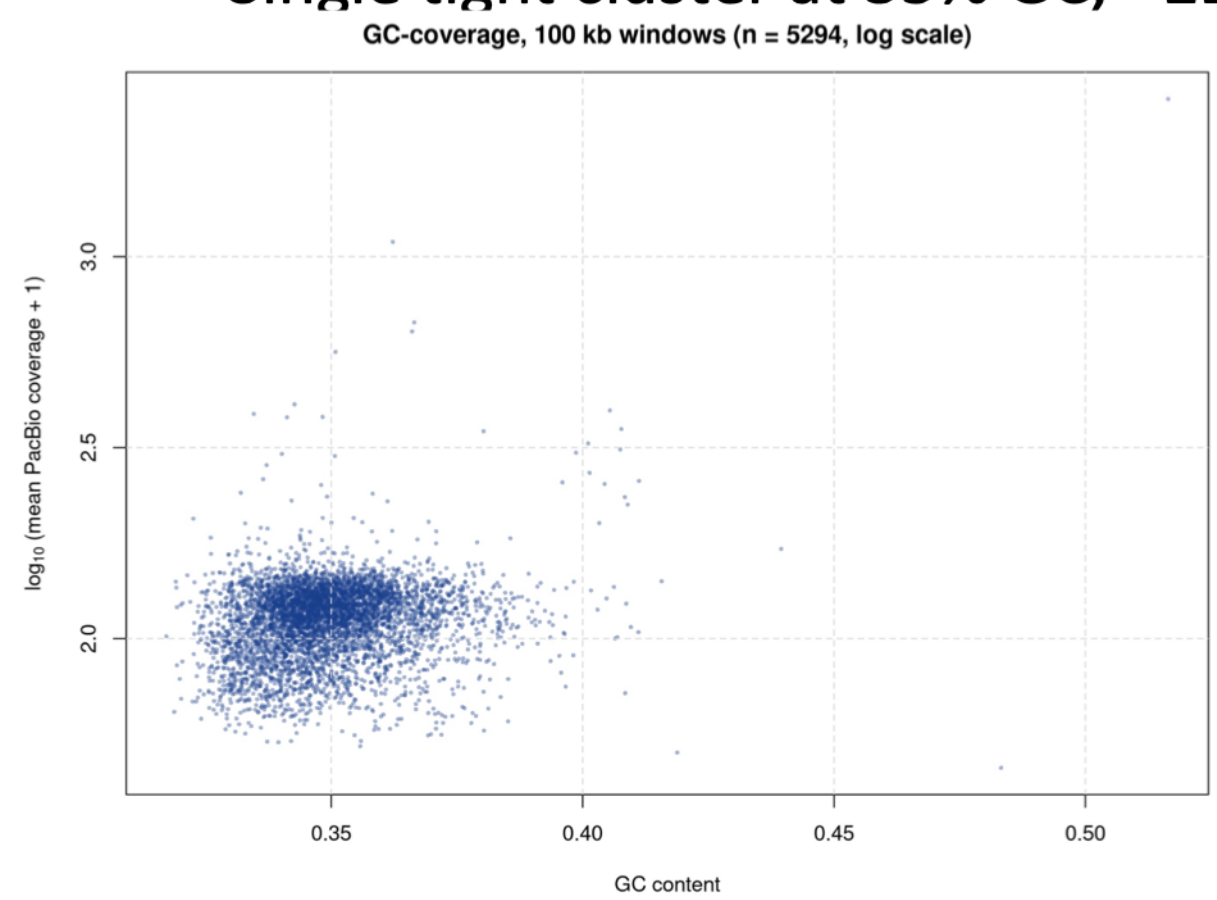

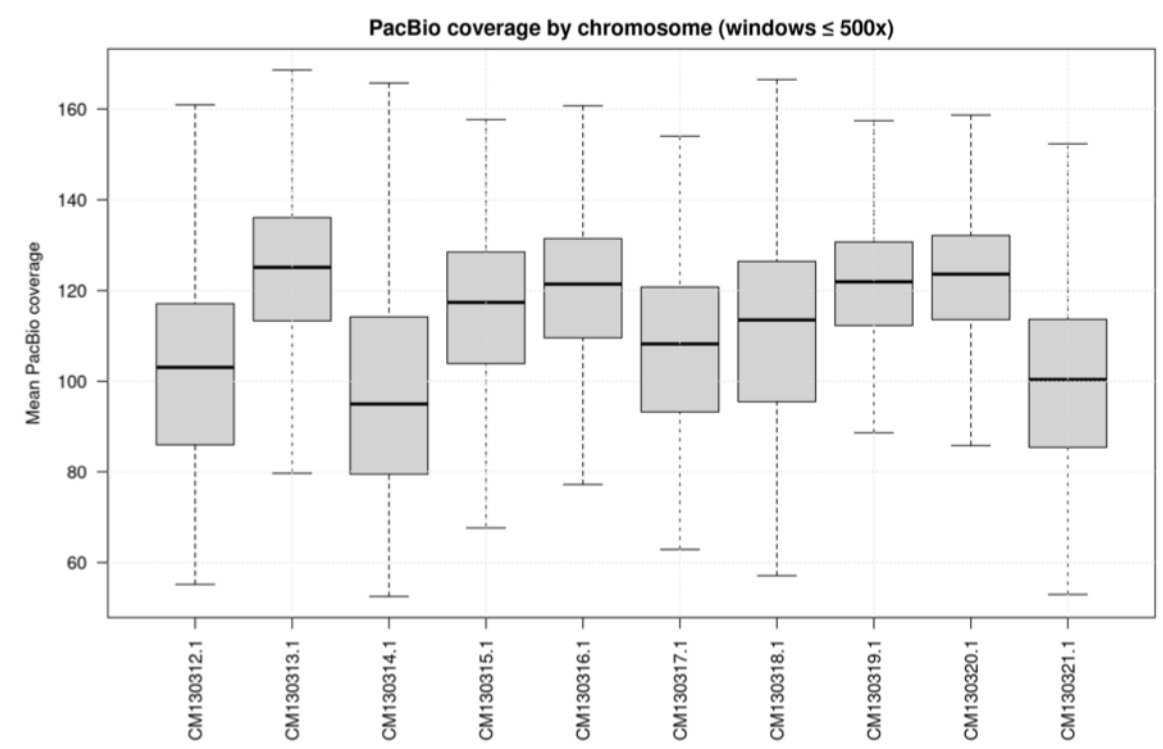

Kraken2 results

- A taxonomic classification tool
- Assigns sequencing reads to organisms by matching k-mers against a reference database
- Crassostrea virginica genome is not in the Kraken2 database
- Oyster reads cannot be correctly classified

Conclusion

- No evidence of contamination in the C. virginica reference genome
